# Memorization of daily routines by children with Down syndrome assisted by a playful virtual environment

**DOI:** 10.1038/s41598-020-60014-5

**Published:** 2020-02-21

**Authors:** Ovidio Lopes da Cruz Netto, Silvia Cristina Martini Rodrigues, Marcus Vasconcelos de Castro, Diego Pereira da Silva, Robson Rodrigues da Silva, Richard Ribeiro Brancato de Souza, Adriana A. Ferreira de Souza, Marcia Aparecida Silva Bissaco

**Affiliations:** 1 Technological Research Center, University of Mogi das Cruzes, Mogi das Cruzes, SP Brazil; 2 Nove de Julho University (UNINOVE), São Paulo, SP Brazil; 3 Postgraduate Programme in Biomedical Engineering, University of Mogi das Cruzes, Mogi das Cruzes, SP Brazil; 4 Professional Master’s program in Health Science and Technology, University of Mogi das Cruzes, Mogi das Cruzes, SP Brazil; 5 Behavior Analysis Core, São Paulo, SP Brazil; 6 Psychology Clinic, University of Mogi das Cruzes, Mogi das Cruzes, SP Brazil

**Keywords:** Psychology and behaviour, Software

## Abstract

A child with Down syndrome, like any other child, may benefit from interacting with memory stimuli, but needs additional support and help. The use of special teaching methods, which add playfulness and use of the computer, can enhance the memory processes of these children. In this work, we present the virtual environment “Nossa Vida (Our Life)”, which was developed to assist children with Down syndrome to memorize action sequences of their daily routine. A daily routine memorization test (DRMT), consisting of a weekly reminder of typical daily routines completed by the children and parents, was performed before (pre-test) and after (post-test) the intervention. The work involved a multidisciplinary team and assessed the effectiveness of the test performed by 30 children with Down syndrome from APAE, a special education school for children with intellectual disabilities in São Paulo, Brazil. The children were separated into two groups (Experimental - GE and Control - GC) with homogeneity and normality of the data. Two hypotheses were tested in this study: H0 and H1, where: H0 = There is no statistical difference between memorizing daily tasks between individuals with Down syndrome who used our ludic virtual environment and those who used the conventional memory method.H1 = There is a difference between the group of subjects with Down Syndrome who used our virtual game environment and the group that did not use it in relation to memorizing the daily task. This produces t = -14.98 and p <0.0001, with H1 being accepted. The results showed that the EG presented significance in relation to the CG and the evolution mean of the children in the EG was 81.82% higher. According to experts (psychologist and pedagogue) from APAE and parents, the playful activities implemented in this virtual environment have been of great interest to children, who had fun, tested hypotheses and questioned them about the sequences of actions performed in their routine daily.

## Introduction

Down syndrome results from a genetic imbalance involving chromosome 21. Also known as Trisomy 21, it is the most common genetic cause of mental disability in humans that can vary from mild to severe.^1–9^.

Its prevalence is of approximately 1 in 700 live births^7,10–13^. According to the last Census of the Brazilian Institute of Geography and Statistics (IBGE), carried out in 2010, approximately 45.6 million people in the Brazilian population (23.9%) have some type of disability, of which about an estimated 300 thousand people were born with Down syndrome^14,15^.

This syndrome causes changes in facial appearance and also compromises children's cognitive ability and physical development^3,4,6,16^. It is usually diagnosed at birth because of dysmorphic facial features that produce a distinct phenotype^17^. The impairment caused in children’s neurodevelopment is observable in psychomotor development and the deficit in many cognitive functions^16^, including difficulties in maintaining attention, deficits in sensory memory and short-term memory, difficulties in the concepts of time and space, delay in language acquisition and memorization of action sequences, changes in language processing that compromise understanding and social exchange^18–20^. These cognitive functions affect learning as well as memorizing specific daily routines^1–4,8^.

Children with Down syndrome have a lower repertoire of functional skills than other children with normal development and may show greater dependence on caregivers’ help. These functional manifestations end up interfering with the ability to perform daily routine tasks^21^. Most of these children also have difficulties in elaborating abstract thinking and absorbing conventions intuitively, such as selecting and performing certain simple functions in their daily life.^2,8,20,22^.

It is also necessary to consider that there is no predictable behavioral pattern for children with Down syndrome, since both intelligence and behavioral history do not depend exclusively on chromosomal changes, and also that environmental stimulation plays a great role.^16^. Therefore, despite having slower reactions compared to other children of the same chronological age, they are unable to memorize sequences and, with training, their above-average memory difficulties can be minimized with the help of environmental stimulation^3^. Special conditions and extra stimulation are needed to learn more time to allow memorization to actually take place^1,3,4,23^. However, the differences between normal and Down syndrome children increase over time if learning difficulties are not overcome or mitigated in a way that does not compromise the development of Down syndrome children.^16^.

Children with Down syndrome are “able to learn and, mainly, to count if they are exposed to conditions appropriate to their needs”^20^. As greater brain plasticity occurs in the first years of life, the development of these children can be improved if conditions and special stimuli are applied in the early years^1^. This optimizes the development of an individual who, under appropriate training, may even have intellectual disability minimized^2^. Therefore, the child with Down syndrome, like any other child, can benefit from interacting with stimuli; however, he / she needs additional support and help.

Individuals with Down syndrome have good visuospatial ability in relation to verbal ability^3,9,19^. According to Lott and Dierssen^19^, morphosyntax, short-term verbal memory and explicit long-term memory of these individuals are impaired, while short-term visuospatial memory, associative learning and implicit long-term memory functions remain preserved.

The strength of these children according to Sainto^6^ is related to visual learning, even having poor visual acuity^6,24^. So, they can benefit from teaching resources that use visual support to work with information^20^, which is confirmed by others authors^5,25^. Yang^5^ mentions that "visual approaches may be more successful than verbal approaches" for people with Down syndrome, while Van Vuren^25^ points out that the use of visual support contribute for minimizing the anxiety and frustration of these children by improving word comprehension and also the memorization of routines and situations. In addition, this author also listed some examples, namely: step-by-step instructions, classroom rules, visual schedules, calendars, among others.

In fact, teachers usually use various techniques in early childhood education, such as elaborate interrogation, self-explanation, summarization and imagery or texts, in which mental and factual images are used to assist in memorizing contents^26,27^ and that can be adapted for daily routines. Other techniques such as conversations, color drawings and photos are also visual resources used by teachers to facilitate the memorization process, a fact explicitly mentioned in Neuro Linguistic Programming (NLP), for which the greater the number of stimuli associated with an information, the greater will be the memorization of this information^28,29^. This fact leads to schools, kindergartens and childcare institutions, such as the Association of Parents and Friends of the Exceptional People (APAE) and the Brazilian Association for Social Assistance and Development (ABADS) to use illustrations related to the sequences of actions performed in classrooms, corridors and bathrooms, among other environments attended by children, as highly accepted techniques of memorization.

Special education experts (psychologist and pedagogue) also believe that the use of special teaching methods, which add playful features and computer use, such as educational or entertainment computer games, serious game, videogame or virtual environment, can enhance children’s memory processes, especially for those with Down Syndrome to assimilate activities of their daily routines.

Nowadays, serious game^30^, videogames, computer games and virtual environments are widely accepted among children and teenagers^31–33^. They are also accepted by teachers who are enthusiastic about the possibility of incorporating digital games into the classroom^34–38^, since some videogames or computer games training can produce a beneficial effects on the brain, which can be observed in cognition, behavior and knowledge acquisition^39^.

Some specific games may provide gains in processing speed, memory and also in attentional, cognitive and social control^39^. These games can improve the short-term visual memory^40^ for completing a task. According to Baddeley^41^ and Pavan and colleagues^40^ this memory reflects the brain’s ability to temporarily retain a small amount of information commonly used to guide learning and/or ongoing actions.

Some authors^35,42,43^ have pointed out to an improvement in student performance when computer-based games were used instead of traditional computer-aided teaching. Whereas positive results also have observed by other authors^35,44–49^, who have used computer-based games (such as virtual environments and serious games) for assisting students with disabilities.

Lanyi and Brown^49^ correlated the learning and fun levels provided by the activity. They noted that learning and its generalization become greater as the child’s level of enjoyment increases when playing a game.

Besides, the enjoyment and pleasure due to ludic context is essential for engagement, participation and motivation in any therapeutic intervention with children, teenagers or adults for teaching or assessment^35–38,43–46,50^. Players act spontaneously when playing games and also use all the knowledge acquired when motivated by a challenge^43–45,51–59^. The effects of playing video games are positive on cognitive, motivational, emotional, and social domains^60^.

However, with exception to “Nossa Vida (Our Life)” software, many other existing educational software, computer games, and virtual environments used to automate only the contents addressed in the classroom^61^. Furthermore, they were not developed taking into consideration the characteristics that motivate their use by children with Down Syndrome^62^. Additionally, they do not portray, or train actions carried out in day-to-day familiar environments such as kitchen, bathroom, bedroom, and living room in a playful manner.

The first version of “Nossa Vida (Our Life)” software was developed by the author himself as part of the requirements for obtaining his master’s degree in biomedical engineering from the University of Mogi das Cruzes^63^. It is a virtual playful environment developed with the objective of helping children with Down Syndrome to memorize important daily routines that occur in familiar environments. However, the utility of this software was verified only through software testing and evaluation of specialists in pedagogy, psychology, and software development.

Developing teaching and monitoring methods that help to promote inclusive education in Brazil is of great value. The inclusive education, which has strengthened after the promulgation of the Guidelines Law and National Education Bases, also known as LDB n. 9394/96, can be considered as framework for inclusion in the country, beyond the Federal Constitution of 1988 that guarantees everyone the right to equality in Article 5o. The resolution CNE/CNB number 2/01, which establishes national guidelines for the special education in basic education, proposes tools to ease the inclusion of special students. Additionally, in Brazil, there are several governmental and nongovernmental associations that assist in the inclusion and development of children with disorders and syndromes. An example is APAE, that currently supports around 250 thousand individuals with Down syndrome, and which also developed various activities such as swimming, volleyball, dance, languages, among others^64–66^.

In this context, our proposal is in line with inclusive education by contributing to the child’s specialized care, assisting family participation, and promoting communication and information. The evaluated and improved computational tool in this research meets this need, providing a further option of information and communication technology for inclusive education and memorization of daily routines. The virtual environment “Nossa Vida (Our Life)”, which was developed in our previous work^63^, was also upgraded thereafter to make it responsive to mobile technology and able to assist the child with Down syndrome for memorizing sequences of actions in familiar environments as well as helping parents and/or education professionals to monitor their progress. The proposed virtual environment will contribute to a child’s capacitation regarding its daily routine that can be replicated to similar environments.

According to the APAE multidisciplinary team who participated in our study, it is a challenge to develop the independence of children with Down syndrome to perform activities of daily living, without which they will not achieve an independent social insertion of the family. Therefore, it is important to find ways to help the child with Down syndrome not to depend on the family for a lifetime, but to be able to acquire autonomy and practice their citizenship instead. In this case, memorizing daily routines is important. Considering this context, we present in this paper the virtual environment “Nossa Vida (Our Life)”, which was developed to assist these children for memorizing action sequences of their daily routine.

## Results and Discussion

During the effectiveness test of our virtual environment “Nossa Vida (Our Life)”, it was possible to observe the evolution of the subjects with Down Syndrome, and the playful characteristics implemented in this virtual environment, which stimulated them for the memorization of daily routines performed in environments similar to those implemented in this virtual environment.

### Effectiveness test of the virtual environment

The Mann–Whitney test proved homogeneity of the sample (*p* = 0.605), while the D’Agostino test confirmed normality of the data obtained from recording the frequency of daily routine actions sequences.

When analyzing these data, the experiment group always presented greater significance, i.e., p ≤ 0.05, whereas in the control group there was the highest proportion of null hypotheses according to the Kruskal–Wallis test.

The paired t’student test (α = 0.05) on the other hand, allows to verify the existence of significance between the before and after in the control group (CG) in relation to the mean values obtained by the participants (EG) when asked about their daily routine.The same test showed that there is a significant difference within the control group before and after the intervention period of the other group, with *t* = −6.69 and *p* < 0.0001. The same occurred for experimental group, where the paired t´student test also proved the significance with *t* = −14.98 and *p* < 0.0001, see Table 1.

**Table 1:** Values obtained with the paired t student test from GC and GE participants data.

	CG	EG
t value	−6.69	−14.99
p value	<0.0001	<0.0001
IC	−0.3071 to −0.1627	−1.1696 to −0.8875

According to results obtained with the Kruskal–Wallis test (α = 0.05), there was also a significant difference between the CG before and the EG after (*p* < 0.0001), the CG versus EG considering only the after (*p* < 0.0001) and EG before and after (*p* < 0.0001), proving the effectiveness of the virtual environment “Nossa Vida (Our Life)”.

Table 2 shows that the amplitude of children’s age was 9 years, corroborating the high experience in the use of educational games.

**Table 2:** Descriptive statistics reporter of children with Down syndrome.

Characteristics	Subitems	Amount (%)
sex/genre	Male	16 (59.26)
	Female	11 (40.74)
Age	10–14	12 (44.44)
	15–19	14 (51.85)
	20–22	1 (3.70)
	Mean (Std. Deviation)	15.(2 (2.88)
Experience with educational games	No	2 (7.40)
	Yes	25 (92.59)

In the case of subjects with Down Syndrome in the EG, 100% of them had evolution when filling the weekly reminder (TMRD), while the subjects with Down Syndrome in the CG did not present any evolution i.e., they did not have a significant improvement in the comparison between the “before and after” mean values obtained in this weekly reminder for each one of the 27 actions described. In Table 3, it is possible to observe the mean frequency of times that all 27 actions available in the TMRD were executed as well as the difference between them (DIF CG and DIF EG).

**Table 3:** Mean of the frequencies that the actions were executed TMRD.

Sequence of Actions available in the TMRD	CG before	CG after	DIF. CG	EG before	EG after	DIF. EG
1) When you enter the bathroom, turn on the light	1.4615	1.5952	0.1337	2.1571	3.0000	0.8429
2) When brushing your teeth, wash your hands before.	0.7253	0.9167	0.1914	1.3714	2.0714	0.7000
3) At the end of brushing your teeth, dry yourself with the towel.	1.4945	1.6429	0.1484	1.1386	2.4643	1.3257
4) Before you leave the bathroom turn off the light.	1.4945	1.5833	0.0888	1.6571	2.4286	0.7715
5) Enter the kitchen to eat	1.8462	2.0833	0.2372	2.4786	3.4286	0.9500
6) When eating an apple, wash it first.	1.0549	1.2381	0.1832	1.6939	2.3929	0.6990
7) When you finish eating an apple, throw the leftovers in the trash.	0.9780	1.2143	0.2363	1.5357	2.2143	0.6786
8) When eating a banana, peel it before.	1.1099	1.3333	0.2234	1.8214	2.4286	0.6071
9) When you finish eating a banana, throw the peel in the trash.	0.9231	1.1905	0.2674	1.5714	2.3571	0.7857
10) Before leaving the kitchen, turn off the light.	1.1758	1.3214	0.1456	2.0357	2.6429	0.6071
11) When you enter the bedroom, turn on the light.	1.8791	2.0813	0.2022	2.7500	3.3214	0.5714
12) When going to sleep, put on your pajamas before.	1.2582	1.3155	0.0572	1.7857	2.3929	0.6071
13) When you get your bed to sleep, take away everything that is on it before.	0.8242	1.1052	0.2810	1.8571	2.4643	0.6071
14) When playing with a puzzle, make more than one puzzle.	0.7253	0.9127	0.1874	1.3214	2.1071	0.7857
15) Before leaving the bedroom, turn off the light.	1.4945	1.5012	0.0067	2.0357	2.6786	0.6429
16) When swimming in a pool, place the buoys first.	0.0000	0.0000	0.0000	1.6242	2.3571	0.7329
17) When you are swimming, eat something	0.0000	0.0000	0.0000	1.3929	2.1071	0.7143
18) Usually swim for a long time.	0.0000	0.0000	0.0000	1.8571	2.5000	0.6429
19) Usually play with objects while swimming.	0.0000	0.0000	0.0000	1.2857	2.1786	0.8929
20) When you leave the pool, the first thing you do is take out the buoys.	0.0000	0.0000	0.0000	1.6214	2.2857	0.6643
21) When you enter the living room, turn on the light.	1.6264	1.6365	0.0102	2.2143	2.7857	0.5714
22) When watching TV, keep pressing the buttons on the remote control before turning on.	1.3000	1.2500	−0.050	1.8965	2.6429	0.7464
23) When you take the remote control of the TV, you already press the button to turn it on.	1.3407	1.3553	0.0146	1.3929	2.3214	0.9286
24) Usually play with objects while swimming.	0.3077	0.4762	0.1685	1.6071	2.3929	0.7857
25) Turn the TV on and off constantly.	1.0696	1.2063	0.1368	1.6286	2.3571	0.7285
26) Change the TV volume frequently.	0.9121	1.0952	0.1832	1.7500	2.3571	0.6071
27) Before leaving the living room, turn off the light.	0.9780	1.5238	0.5458	1.8214	2.4286	0.6071
Mean DIF (before and after).			0.1332			0.7334

For example, the mean of the difference for the values of CG regarding the 27 actions performed before and after (the use of our virtual environment) is equal to 0.1332, while the mean difference related to before and after of EG is equal to 0.7334, i.e., the mean of evolution of the subjects with Down Syndrome who interacted with our virtual environment “Nossa Vida (Our Life)” was 81.82% higher.

Regarding the subjects’ behavioral record, it was possible to verify that 85% of the subjects with Down Syndrome have a computer or any other device that can run the virtual environment “Nossa Vida (Our Life)” at home. Among these subjects, those who do not play at home reported that they do not have many games and/or virtual environments of their interest.

**Figure 1:**
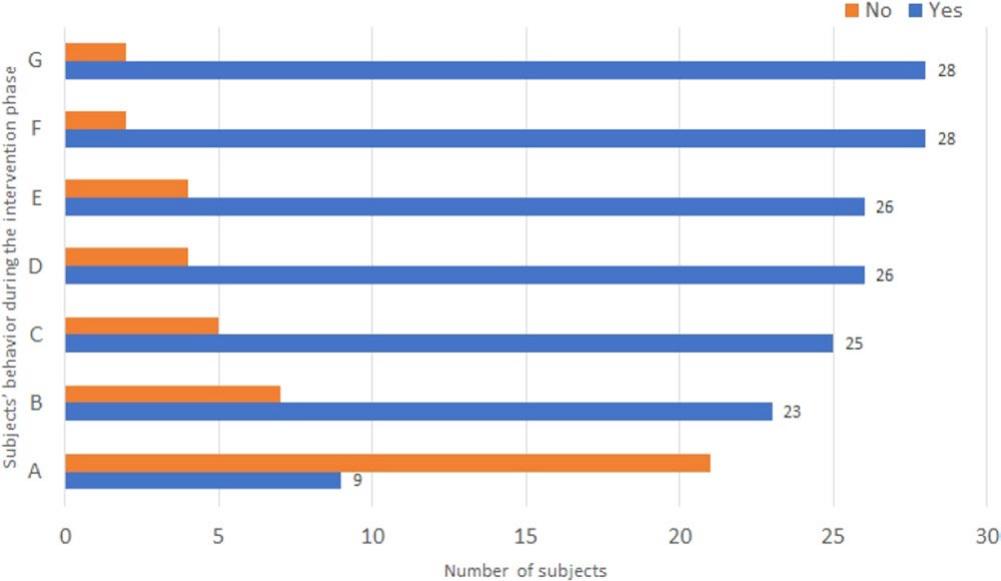
Subjects’ behavior during the intervention phase: (**A**) Requested help to start the virtual environment; (**B**) Requested help to use the virtual environment; (**C**) Requested extension of pre-established time; (**D**) Played without stopping; (**E**) Verbalized positively about the game; (**F**) Learned to use controls easily; (**G**) Wanted to play again.

When analyzing the behavior of the subjects with Down Syndrome, one should always analyze their features and reactions^52^. During the behavioral recording of the subjects with Down Syndrome who interacted with the virtual environment “Nossa Vida (Our Life)” it was observed that they presented very positive behaviors during this interaction as illustrated in Fig. 1.

In this chart it can be seen that the kids liked the virtual environment “Nossa Vida (Our Life)”, once most of them asked for an extension of time to continue playing, played without stopping, learned to use the controls easily, and wanted to play again. This demonstrates that our virtual environment was accepted by the target audience and reached the goal for which it was codified.

According to the literature, whenever there is an affinity between the subjects with Down syndrome and the person who assists them during the intervention with the virtual environment such as their parents or teachers, the results are more positive^67,68^. In this case, the subjects with Down syndrome showed behaviors such as establishing limits and affection, following the rules consistently during the time of the intervention. In case of the subjects with Down syndrome enrolled in this study, the improved behavior was possible due to great affinity between the subjects and their IT Teacher.

### Updating the virtual environment

The results and feedbacks obtained during the effectiveness test performed by children with Down syndrome and the expert’s evaluation led us to implement some improvements (upgrade) in our virtual environment, resulting in “Nossa Vida (Our Life) 2.0”, which are highlighted in the following paragraphs.

This new version gains the possibility of being responsive and the capability of running on any device, because it is multiplatform compliant, i.e., on a smartphones, tablets, notebooks, and computers. Besides, many children can use it concomitantly. It was implemented a user-friendly graphical interface in two dimensions, (2D) using pictures, images, or icons for representing characters and scenarios^69^.

It was also created a module for monitoring a child’s evolution, which records the actions (i.e., the decision-making) of the child while playing. To enable this, an access control has also been coded in the virtual environment, which allows children, their guardians and professionals from special education institutions such as APAE to access only the modules of their interest. While children access the virtual environment just to play, their guardians and professionals can follow their actions in the environment.

Further, it allows a greater interaction of the user who can alter the bottom images of all environments. Besides, there is a screen for the characters’ personalization, and after the defined choice, it is possible to take a picture generating the instantaneous download of that image.

A playground where it was possible to interact with the two characters of the game in a seesaw, swing, and slide was also created. A parrot was placed in the rooms of the house to perform the role of a mascot and help in some game actions.

The user of the virtual environment “Nossa Vida” can execute it by freely and randomly selecting the environments he wants to explore, as well as proceeding inside each environment in a fixed sequence; however, with the possibility of leaving without the completion of the sequence of necessary actions. Furthermore, as implemented in the first version, it also comes with a possibility of following a logical sequence of actions usually accomplished by a child as in a daily routine at his residence and afterwards can make the transitions among the sceneries that he wishes to, through the main scenery acted by the interface with the image of the segmented house. Therefore, the child will be able to access the environment (phases) following an execution order of any number of times he wants.

The user’s interaction with the virtual environment is accomplished in first person through the use of mouse for devices such as desktop and notebook as in the first version^61^, as well as using touchscreen technology for mobile devices such as smartphone and tablets. According to APAE professionals, the use of the mouse is also adapted for children with Down syndrome in addition to touchscreen technology, which is currently the most usual form of interaction^70^.

**Figure 2:**
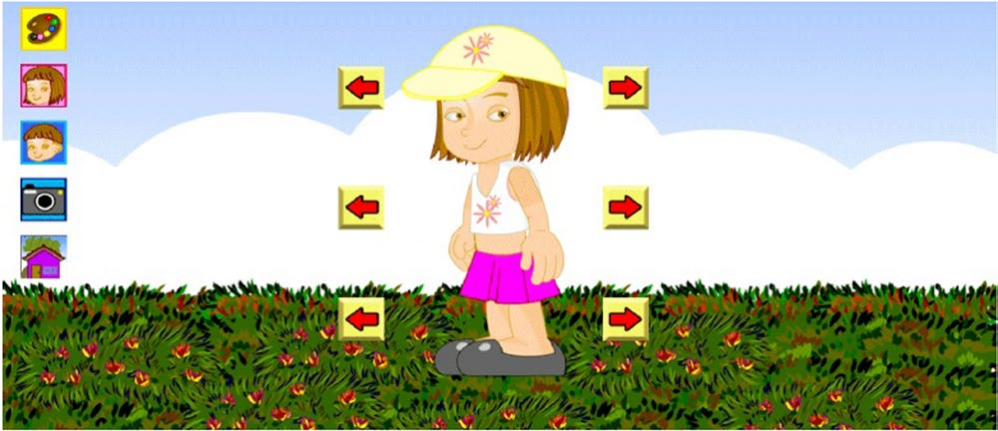
Interface that allows the characterization of the character and the environment.

The main functionalities of this virtual environment are presented, detailing the actions and answers, and showing new illustrations. A female character will be used to detail the features. The logic is identical for the male character. When starting the game, the ludic opening screen is presented. The text is presented with its verbal reading. Further, the user can choose the female or male character and customize it (Fig. 2) by choosing its clothes, accessories, and environments. They can also take photos of their visual, obtaining the it through a PNG file instantly, which can be saved with a name chosen by the player/user.

**Figure 3:**
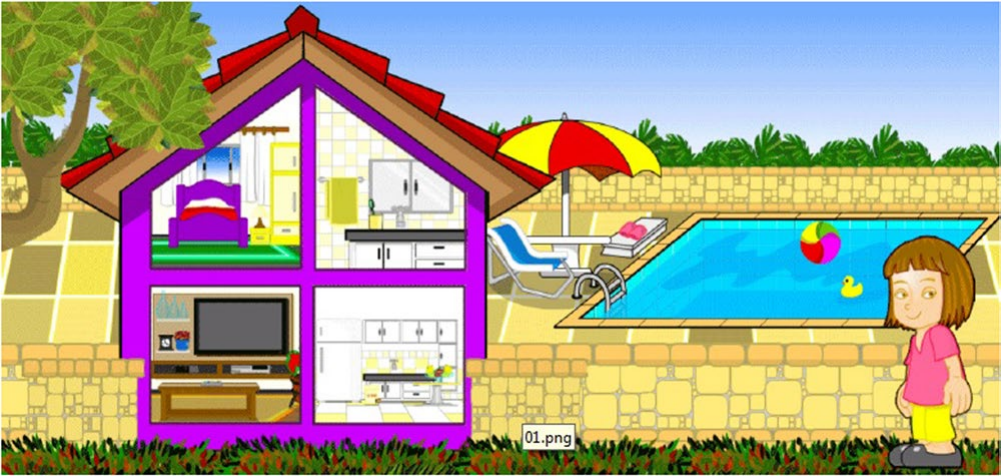
Interface in which the user will choose the environment and the initial activities to be developed.

After the character has been characterized, the initial navigation interface in which the environments that the user wishes to explore will be chosen (Fig. 3).

**Figure 4:**
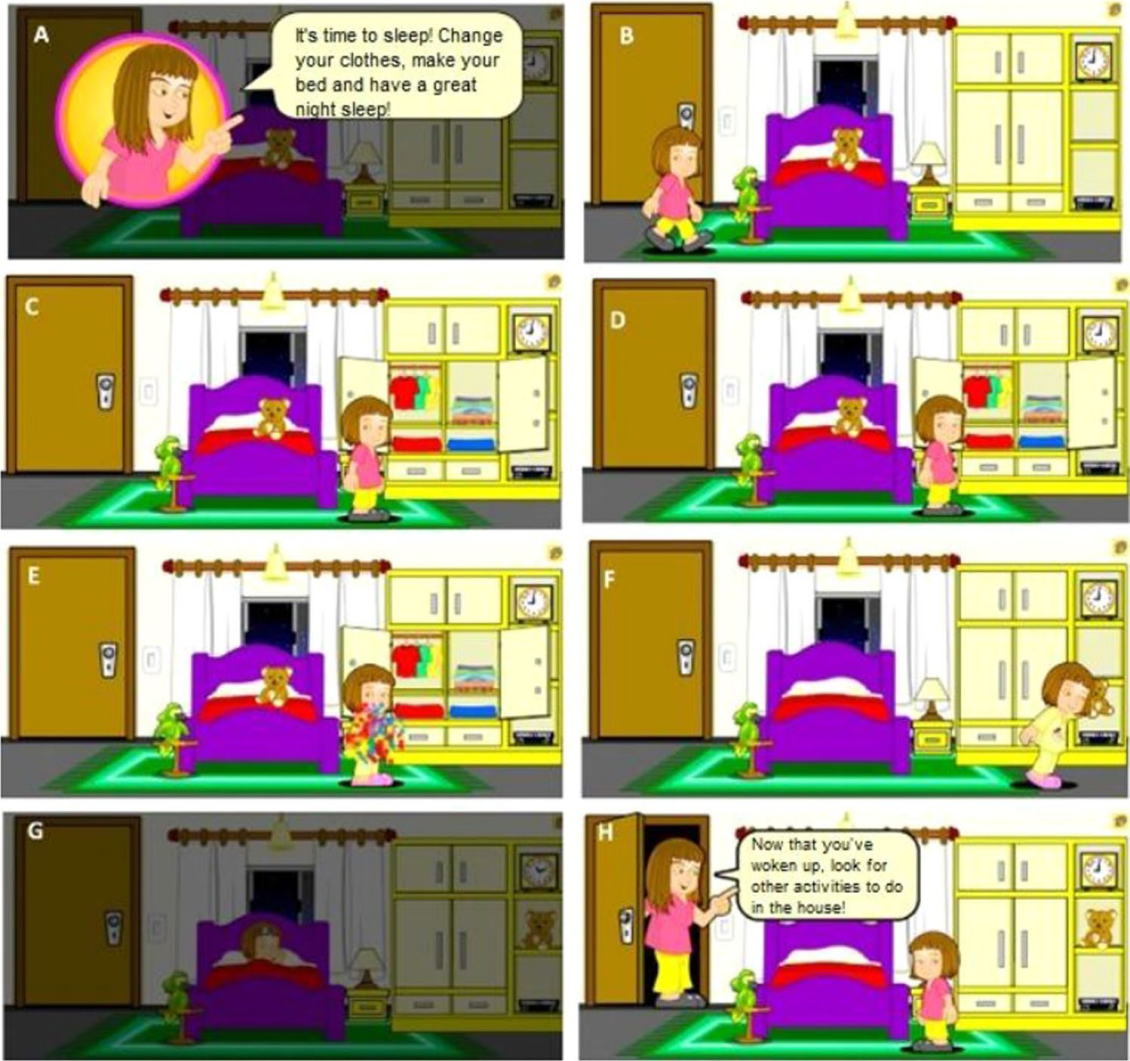
Room Routine: (**A**) Interface with the sequence of instructions that will be performed; (**B**) Interface where the user can choose which interaction will be performed first; (**C**) Interface exposing the open wardrobe where the user can choose clothing; (**D**) Interface that shows the user’s change of clothes; (**E**) Scene showing the user putting the stuffed animal in the closet; (**F**) Interface showing the user sleeping; (**G**) Mother feedback screen.

Once the environment has been selected, its exploration is begun from a sequence of tasks. For example, an environment can be selected wherein the user can play with a puzzle and perform the whole sleeping routine, which involves cleaning the room by keeping the stuffed animal in the wardrobe and putting on suitable bedding. At the beginning of each environment, the mother appears giving directions of the sequences that will be performed in that environment as shown in Fig. 4.

A key point to be highlighted is that the environment is kept in a cheerful and affectionate state through a mascot in the form of a friendly parrot, which helps by giving a sound feedback in all environments/scenarios of the virtual environment “Nossa Vida (Our Life)”.

Our virtual environment can be accessed at the website www.ovidio.eng.br/nossavida. The player trail in this environment is recorded in a database and presented in report form, which can be accessed at http://nossavida.azurewebsites.net using the same login and password created in the game.

## Discussion

The interface greatly influences the accessibility of the computer and the engagement of the user. A variety of media such as text, images, sounds, and movies can be used, allowing disabled users to also operate computers easily^71,72^. The entertainment industry uses these stimuli to increase user motivation and to encourage them to continue to play despite the frustration of losing^73^. This factor is important for all students (children, teenagers, adolescents or adults). including those with special needs^30,49^. Therefore, we take this into account when designing and implementing all the elements of the graphical interfaces of our virtual environment.

The projected 2D graphic interfaces and interactivity mechanisms encoded in the new version of this virtual environment continuing to provide a playful environment for a user to focus his attention with enthusiasm on performing the action sequences of each home environment, of which some are enhanced with additional features. Interfaces are colorful and contain children’s everyday objects, which often awaken their natural curiosity and help immerse them in the plot. The plots involve the sequences of daily actions that take place in a home environment. The visual elements that make up the interface were developed to attract the selective attention of the users directing the focus to game and avoiding distractions from external elements. That is, we used figures that can awaken their natural curiosity and help immerse them in the learning of daily routine.

The sound alerts and other feedbacks (as images and writings), which are executed in response to the user’s decision-making (and that correspond or not to the expected behavior), observed through the intervention performed with the first version, indeed corroborate to an immersion of the users in the virtual environment. The fact that the user could choose the gender of the character (female or male) and having five environments of the house to explore were also the factors that really motivated the subjects with Down syndrome who participated in the effectiveness test. This was because the implemented environments were very familiar to the subjects.

The characters were designed in a cartoon style because cartoons, according to literature, are an efficient resource to communicate something to the children. “The colored moving images first capture children’s attention before they understand any message, providing them a world full of symbols that reflect complex concepts to explain to a young child”^74,75^. In other words, comic-style figures were used to provide children with enjoyment associated with informative content.

The upgrade also added suggestions made by the experts (psychologist, pedagogue and Games Specialist) who evaluated the first version, as well as suggestions from the subjects with Down syndrome themselves and their parents. This made the “Nossa Vida (Our Life)” virtual environment more attractive and with a greater potential for immersion of the subjects with Down syndrome as well as a tool of utility even for the professional who assists these subjects, according to a new evaluation realized with specialists in games. In the new version, the characters were redesigned to possess a modern look and additional elements were added, such as a mascot (i.e., a parrot), more TV channels, sound effects, wallpapers, personalization of the character, playground environment, database for storing the events realized by the user, report issuance, and graphs visualization.

Sound alerts were inserted at the entry points of all environments as well as descriptions of all actions to be performed by the users in the environment narrated by the mascot. The personalization of an environment’s wallpapers and characters; although the last one can be realized only to allow the user to take a picture and keep it as a souvenir; are also the elements that, according to the psychologists and pedagogues who participated in the project, retain the attention of subjects with Down syndrome. The tests of use quality (usability and functionality) which were performed for each of the environments that make up the new version of the virtual environment “Nossa Vida (Our Life)”, allowed not only to verify the actions sequences, but also the feedback implemented performed as expected, which is presented in sonorous, graphic and written form to assist the player in the room, kitchen, bedroom, bathroom, yard and playground.

The virtual environment “Nossa Vida (Our Life)” allowed the subjects with Down syndrome to begin the game by a fixed sequence of the environments running with a sequence of actions pre-established in each environment, as implemented in the first version, or by a free-running of the environments/scenarios with fixed action sequences only in each environment as implemented in the current version. Thus, the environment enabled the subjects with Down syndrome to create skills in all environments. Therefore, when entering the game/virtual environment it is possible to choose the fixed or the free routine; remembering that in the free routine, the entire way trailed by the user is registered in the database.

In the free-running mode, the goal of the user is to decide what he/she wants to do within the virtual environment “Nossa Vida (Our Life)” v.2.0. However, through the reports generated by the virtual environment itself from the automatic recording of the user’s actions (i.e., all their steps) within this environment, it was possible for parents or professionals to take a decision and monitor their progress; this being very important for the learning evolution of the subjects with Down syndrome^10^.

The virtual environment “Nossa Vida (Our Life)” v.2.0 is at par with software found in the literature or commercial environment, both educational and entertainment, considering the important requirements such as graphical interface, interaction mechanisms, feedback, attractiveness, usability, gameplay, and mainly accessibility over the Internet. Compared to other similar products i.e., educational software developed specifically for children with Down syndrome such as the “Jecripe” of Federal University Fluminense^76^ and “O Trapalhão” developed by Vogel^46^, the usual software tests and the effectiveness test performed with the help of children with Down syndrome met expectations, once the assumptions were met.

According to experts (psychologist, pedagogue and in games specialist), “Nossa Vida (Our Life)” v.2.0 continues to serve all assumptions of the software above, in addition to having characteristics that differentiate it from those such as the possibility of registering all the way traveled, the simulation of a house, bringing reality and the fact of being responsive, and running on any device. It allows the subjects with Down syndrome, especially children, to identify themselves directly with the main character, which was modeled to present the physical characteristics of these children, which was only implemented in JECRIPE only. Further, motivating the execution of sequences of daily routines actions that occur in familiar environments such as living room, kitchen, bathroom, bedroom and a house in which the yard has a swimming pool, and next to a square with playground was not observed in any of the evaluated software.

Although children using the developed virtual environment do not have a pool in their own backyard, they can demonstrate their knowledge and/or learn from the correct sequence of actions to use a pool in a public or private club or family and friends’ homes. This is particularly important as a way to prevent drowning accidents in swimming pools^77^. Therefore, the swimming pool was implemented in the garden of our virtual environment in order to promote childhood water safety^77,78^. According to the American Academy of Pediatrics^78^ “drowning is a leading cause of injury-related death in children”. Every year in Brazil, more than 1,100 children die of drowning. In 2011, according to information obtained from DATASUS, a database of the Unified Health System, 1,115 children aged 0 to 14 years died and 293 were hospitalized. Additionally, the death in children due drowning is more often in weekend^79^.

It is worth mentioning that the major contribution of this research is the upgrade carried out in the virtual environment “Nossa Vida (Our Life)”, which helps the professionals of the multidisciplinary team to diagnose and/or follow-up the process of making the bjects with Down syndrome memorize the sequences of actions implemented in the environments, because the registration of the actions (i.e., decision-making) of the subjects with Down syndrome in a database is carried out automatically by the virtual environment as soon as it begins execution.

This record waived the presence of an evaluator during intervention or diagnostic procedures, allowing the subjects with Down syndrome to act more spontaneously^46^. According to the literature^43–45,51–59^, children act spontaneously when playing as well as using all the knowledge already acquired. Additionally, the reports and graphics that can be generated by the environment from the recorded data allow us to conclude whether the subject with Down syndrome has actually established a routine. It may also be able to minimize some of the emotional conflicts mentioned by^80–82^ that occur between family members, health care staff, and support staff; conflicts that end up hampering or even distorting the work of the health care team.

The scientific challenge was, therefore, to come up with an important problem for the Special Education area and to develop a solution based on playful resources; the virtual environment “Nossa Vida (Our Life)”. On the other hand, the technological challenge was to make this virtual environment responsive, since there was a technological advance that prevented its enrichment after the implementation of the first version. The technology used earlier i.e. Flash, is currently not used for game development because it does not run on smartphones and major browsers.

The playful activities presented by the virtual environment, according to the feedback from the parents of the subjects, provided great interest to subjects with Down syndrome who were enrolled in this study. The subjects had fun, tested hypotheses, and questioned their parents about the sequences of actions performed daily.

Analysis, design, and implementation of new functionalities in accordance with the feedback obtained from this effectiveness test and the evaluations made by experts in games were performed, which resulted in an upgrade that made this virtual environment responsive and with more attractive elements for the subjects with Down syndrome and professionals in the area of inclusive education. It presented a new environment represented by the playground, customization of the character to undertake photography, more wallpapers for customization of the environments, more TV channels, registration of actions of the user in the database in the environments, generation of graphics from this stored data, and access control of the users.

The advantage of being responsive is that the virtual environment “Nossa Vida (Our Life)” can be executed online on several computer platforms and even on mobile phones or tablets. This is important because the number of smartphones surpassed those of computers, becoming Brazilians’ favorite devices to connect to the internet in 2014, as can be observed in the National Household Sample Survey (PNAD) conducted by the Brazilian Institute of Geography and Statistics (IBGE). In addition, cell phones are taking up more and more space within classrooms: in 2016, 52% of schools were using these devices in student learning activities as pointed out by the “ICT Education 2016” survey conducted by the Center for Studies on Information and Communication Technologies (CETIC).

The software tests performed in the virtual environment “Nossa Vida (Our Life)” by the author and the anonymous evaluation performed by specialists were also positive, demonstrating that the functional, non-functional, educational, and psychological requirements are sufficient and necessary; and are working as expected.

An innovative “digital” approach was presented through a playful and responsive virtual environment that works with specific limits of subjects with Down syndrome, enhancing the memory of sequences of actions performed in a family’s daily life.

It is important to emphasize that, in the present study, we did not intend to bring gender stereotypes of how boys and girls should behave or do something different in our virtual environment. Instead, we wanted only to provide them with an equal opportunity to act in our virtual environment through a character of your free choice, having female or male characteristics. A character was implemented taking into account some colors and objects, which according to the literature^62,83–86^ boys and girls usually express preference. However, who decides which character will act in the virtual environment are the children themselves.

We had in mind only to create characters with whom the children could identify and thus to promote the immersion and memorization of the same daily routines. That is, we expect both boys and girls to fully exploit their potential and talent. They can play with a character chosen by themselves, and that they liked and/or identified themselves more. We believe that by allowing this choice, we are promoting equality. If a girl prefers, she can play with the male or female character and the same happens for the boy.

If the child plays without adult supervision, he/she will probably play with the character (male or female) of his/her preference. However, in order to know which character both boys and girls like more or have preference to play in the virtual environment “Nossa Vida (Our Life)”, it is necessary to carry out a future study, in which the trace of the children participating in the study within the virtual environment must be analyzed.

Online learning should be used together with an educational strategy rather than in isolation^87^. As mentioned by^88^, collaborative participation between teachers and users should be taken into account when developing support materials (such as games, software, devices, and assistive technology).

Taking this into account, it is worth mentioning that the virtual environment “Nossa Vida (Our Life)” was developed to be used as an auxiliary tool to an educational strategy, as a way of maintaining the learning that was already being carried out in the school environment of the APAE. Therefore, the effectiveness of this virtual environment was verified in this context, counting on the monitoring of this learning by a qualified professional.

As supervised teaching has already been done with psychological observation, we believe that the virtual environment “Nossa Vida (Our Life)” can be used alone for the maintenance of learning. This virtual environment was also evaluated by experts in psychology and pedagogy before the effectiveness test with children, and it was also approved by the multidisciplinary team of the APAE, where the children with Down syndrome and their parents were invited to participate of the study. Therefore, it can be used by children with and without advice of an adult like their parents or APAE multidisciplinary team, because it only promotes the learning of good practices regarding the daily routines in the home and for safety in the pool water.

The presence of a tutor is not necessary when the child uses it, since it may also cause inhibition^46^. However, parents and/or APAE multidisciplinary team can have access to the child’s trail within our virtual environment (version 2.0) and, in this way, see if he/she is creating a habit and demonstrating autonomy. According to Mara^89^, automate skills through practice make them not only acquired mentally, but also learned and easily accessible, providing support for more complex activities. On the other hand, Matovu and colleagues^90^ recently pointed out that teachers should remain close to students with learning disabilities when classroom interventions occur because the relationship between teacher and student contributes to a statistically significant improvement in student learning.

Any child with or without disabilities can use the virtual environment “Nossa Vida (Our Life)” in- and outside of preschool setting as long as she/he wishes to explore all implemented scenarios and functions, which can take hours and even days. However, depending on her/his abilities with the computer, familiarity with this virtual environment and, mainly, the motivation to play, the child can finish the execution of our environment in a minimum time of 11  minutes.

Children with or without disabilities, regardless of gender and ethnicity, can use the virtual environment developed, but in the first version we created characters that present physical characteristics exclusive from children with Down syndrome. This was done in order to promote their immersion in the virtual environment. However, in future work we also intend to create more characters aiming to embrace diversity, considering personal or cultural identity, to really facilitate the immersion of more children with or without disabilities. We intend to create, for example, characters black, Japanese, Indian, among others, implementing characteristics that take into account children’s emotional factors in a positive way, which according to CRPD “are important for the development of inclusive schools and have a great impact on students’ learning”^91,92^.

It´s known that the cognitive formation is built during the individual´s lifetime and ludic and plays are elements of great influence in the articulation of his mental mechanisms. Therefore, games consolidate mental schemes that are already formed, and they also provide pleasure and/or emotional balance, stimulating the intellectual autonomy growth. They can also provide the exercise of thinking and it is understood as an intentional and imaginary human act, which aims to change the present and the reality although doing it respecting the rules^93^.

Another important factor to emphasize is that when a stimulus is constantly presented, its potential for behavioral reinforcement magnitude decreases^94,95^. Hence, as computer access and use has now become widespread, one might consider it to have become a low-magnitude behavioral reinforcement. On the other hand, our virtual environment due to all the implemented features, provides novelty for children with Down syndrome, presenting greater magnitude of reinforcement.

Additionally, Rose^96^ performed an experiment using a simple sensorimotor task and found that, in fact, there is a transfer from the virtual to the real world, when the arranged elements approach each other in similarity. The authors obtained robust data on this transfer and concluded that while the organizational set applied in both real-world and virtual-world learning may be similar to generate high levels of training transfer, the cognitive loads associated with every operation may differ in both situations considered. On the other hand, West^97^ noted that training using the video game has an effect on the player’s hippocampal system and that this effect may be beneficial or detrimental depending on the navigation strategy and the genre of the game used. The authors observed that strategies involving increased spatial memory increased hippocampal gray matter^39,97^.

By using games and play, children can appropriate resources that develop learning, which includes children with intellectual disabilities. According to Emília Ferreiro^98^, the interruption of psychogenetics and the lack of appropriate skills can hinder the acquisition of knowledge and, later, the social life of these children. Considering the comments above and the results obtained, we point out that the virtual environment “Our Life” presents the characteristics necessary to develop the knowledge of children with Down syndrome, as well as improve their adaptation to daily routines and implement new knowledge appropriate to a gradual evolution of their emotional and intellectual maturity.

In summary, it’s believed that the virtual environment developed is not just for children’s entertainment, but rather a tool to help teachers in the process of the children’s learning and its maintenance, and also assist them to participate in society by encouraging the development of their skills. This study had in mind the desire to contribute to a quality and inclusive education, considering what CRPD (in its article 24, page 7) says, that is, we have the moral and legal responsibility to build systems that support all students to achieve “ the full development of human potential and sense of dignity and self-worth, and the strengthening of respect for human rights, fundamental freedoms and human diversity”^91,92^.

## Conclusion

The effectiveness test of the virtual environment “Nossa Vida (Our Life)” was performed by children with DS from an APAE of the São Paulo city, Brazil. The results showed significance in the sequences of actions performed by the experimental group (EG) in relation to the control group (CG), which were recorded by the parents in the weekly reminder of their daily routine. That is, the mean evolution of the children (from EG) who interacted with the virtual environment “Our Life” was 81.82% higher.

According to their parents’ reports, the playful activities implemented in this virtual environment have promoted great interest on the children, who had fun, tested hypotheses and questioned them about their own sequences of actions performed daily. This result is in agreement with the literature, which mentions that customized virtual environments in fun games format improve the learning of children with disabilities since they seem to motivate their engagement.

Since the virtual environment “Nossa Vida (Our Life)” has the function of promoting learning when the child uses it, the assistance of an adult might be inhibiting. On the other hand, parents and/or APAE multidisciplinary team can have access to the child’s trail within this virtual environment (version 2.0) and, in this way, see if he/she is creating a habit.

This virtual environment can be used by children with and without advice of an adult (their parents or APAE multidisciplinary team), because it only promotes the learning of good practices regarding the daily routines in the home and for safety in the swimming pool. Besides, it was evaluated by experts in psychology and pedagogy before the effectiveness test with children. Besides, it was also approved by the multidisciplinary team of APAE, in which the children with DS and their parents were invited to participate of our study.

The criteria for selecting the “daily routines” to be taught and the environments implemented were established after contacting experts in special education (psychologists and pedagogues) who worked at APAEs. Among the several daily routines that could be trained, some of the ones that a child performs in his own home were selected in environments such as kitchen, bathroom, living room, bedroom and backyard, because according to these experts consulted, it could represent for a child with DS a continuity at home to what is already worked by the kindergarten professionals of support institutions like APAE, for example.

In fact, the daily routines were identified by APAE professionals, who claimed that the learning acquired at school was sometimes lost in the family environment. Thus, the developed virtual environment could act as a self-monitoring strategy for children during out-of-school time. This tool aims to reinforce the learning acquired in this institution.

As a future work, we plan to test virtual environment “Nossa Vida (Our Life)” with other disabled children such as those with Autism Spectrum Disorder and also nondisabled children because learn memorization techniques is important for them too. Besides, we believe that people with Alzheimer can also benefit from our learning tool. Additionally, we intend to increase items for character customization as occurs in entertaining games for player immersion, and also to build new scenarios that can be customized for each social reality of a child with Down syndrome as well as sequence of actions that are important for children’s awareness, self-care and autonomy, taking into account situations not only inside the house, but also in the school and in other environments as, for example, in clubs (with several swimming pools) and also in the beach.

## Materials and Methods

### Ethical aspects, participants and effectiveness test protocols

The study was conducted under the approval of the Ethics Committee in Research involving humans at the University of Mogi das Cruzes (CAAE 39466714.5.0000.5497, number 927.118) on December 15, 2014. A written informed consent was signed by the guardians on behalf of the subjects with Down Syndrome enrolled in the study after they had been adequately informed about the aims, anticipated benefits, methodology of the study, about the privacy of research subjects and confidentiality of their personal information. All specialists also provided a written informed consent at the time of enrollment. This assessment was carried out anonymously to not violate the ethical principles, as observed in resolution 510/16 of the National Health Council, Health Ministry, Brazil.

To perform the effectiveness test of the “Nossa Vida (Our Life)” virtual environment, 30 subjects with Down Syndrome aged between 10 and 22 years old (see Table 2 shown earlier), who were studying at an APAE (a special education school for children with intellectual disability) from a city in the São Paulo Metropolitan Region, state of São Paulo, Brazil were chosen.

According to the information supplied by APAE multidisciplinary team, all research subjects had: the same socioeconomic level, i.e, all children enrolled in this study were from low-SES families; their homes near the school; the same learning opportunities; computer literacy; already used computerized game in the computer classes of school.

The sample size is limited to the total number of subjects attended at the APAE unit involved with our study. All subjects met the inclusion criteria mentioned above. Additionally, the central limit theorem, which is often applied in Biomedical Engineering, also contemplates this sample size.

These subjects were then separated into 2 (two) homogeneous groups by using simple random sampling, considering the average age, the time they attended APAE, and schooling. The groups were denoted as experimental group (EG) and control group (CG).

The subjects of the EG group, with a mean age of 15.1 participated in all stages of the developed virtual environment, while the subjects in the CG, with a mean age of 15.2 did not use the environment in the first stage of the search.

**Figure 5:**
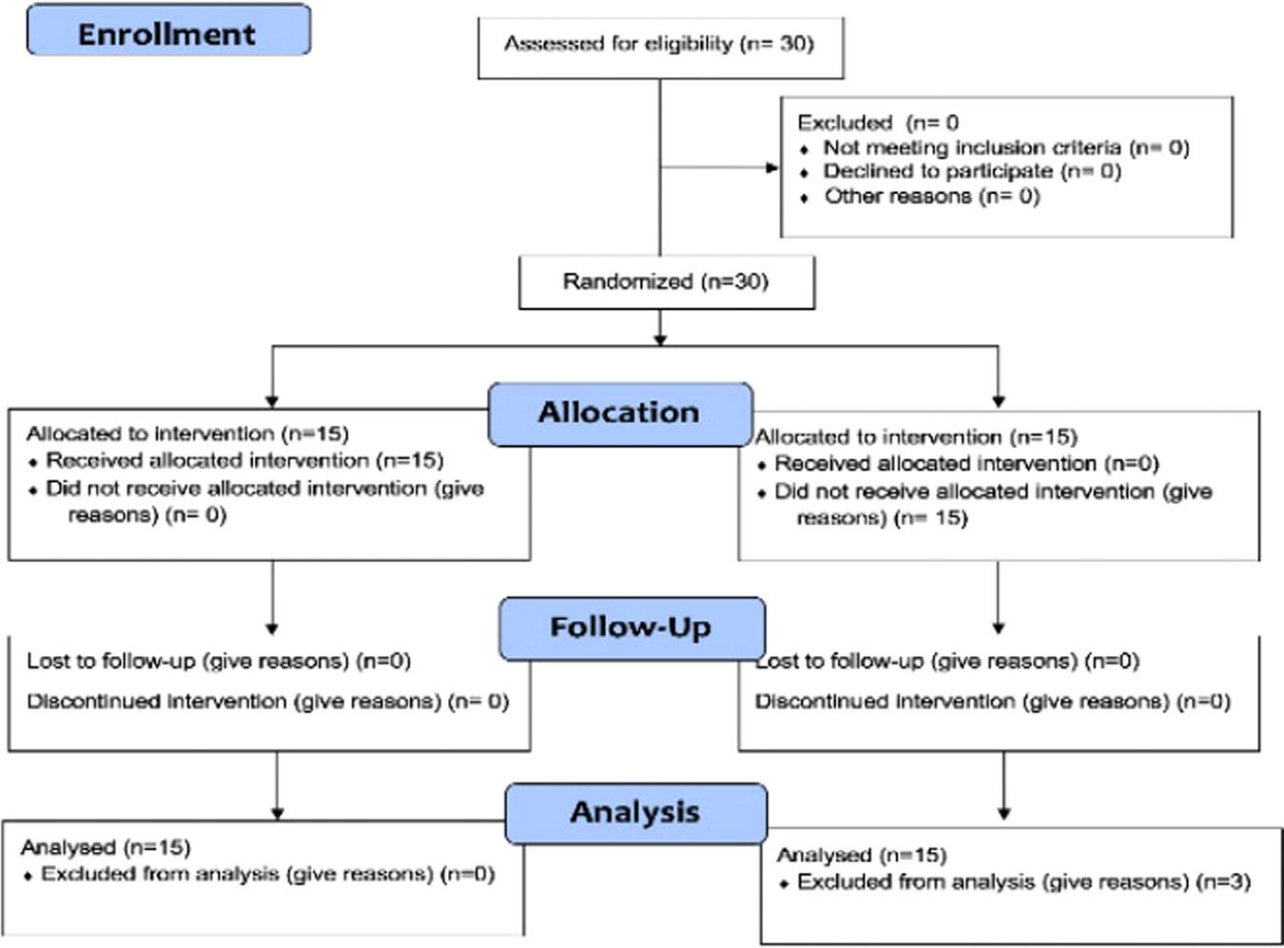
Flow diagram-Consort and Trend.

The number of subjects and protocols employed in this study are shown in the flow diagram of Fig. 5 and described below.

After the research presentation, it was obtained the signature of the children’s guardian in a written informed consent. After that, the dates for the pretest, intervention, and post-test were agreed between the multidisciplinary team of the APAE and the guardians on behalf of the subjects, who were enrolled in our study.

The pretest was applied to subjects using the Daily Routine Memorization Test (DRMT), an instrument for data collection developed by the authors with the aid of a psychologist. It is a weekly reminder of typical daily household routines to be completed by the children and their parents (see Supplementary Material I and Table 3 shown earlier). It was applied before (pre-test) and also after (post-test) intervention with the virtual environment “Nossa Vida (Our Life)”.

The time they actually spent to fill out the DRMT form was not counted neither before nor after intervention period. In fact, it was important to record just how often each action listed on this form was performed for one week, that is, from Monday to Sunday. Ideally, this frequency should be recorded as soon as the action occurs.

Regarding the time spent in performing the intervention with the virtual environment “Nossa Vida (Our Life)”, the time spent by children to finish each phase, ie, to execute the sequences of actions in the virtual environments related to the kitchen, bedroom, living room, bathroom and yard with swimming pool, was not counted as well. In our study, the memorization process is more important than the time that it takes. We only gave them a total time that would be enough for them to complete our game, according to information about their performance in games already used at APAE. The multidisciplinary team of the APAE kindly inserted this time to play game into the children’s routine in a way that all children used our virtual environment.

In the upgrade performed in our virtual environment “Nossa Vida (Our Life) v.1.0” as a function of the results obtained from effectiveness test, an algorithm has been implemented for allowing parents and professionals to know the decision making of the children in the environment, as well as the time they have remained in each scenario and action or sequence of actions.

Both groups of traditional teaching of memorization of daily tasks performed a post-test, in which the TMRD was reapplied.

During the intervention phase, the subjects were taken from one classroom to another, being aware that they would play and use a computer game. The time of application of the virtual environment embraced a total of 500  minutes, having ten sessions of 50  minutes, and these were applied twice a week throughout 5 weeks. Additionally, the children continued their traditional activities at APAE.

TMRD was performed by the subjects’ parents for seven days in a row, in both pre-test and post-test i.e., the period of one week was considered to understand what the subject usually performed before the intervention with the virtual environment “Nossa Vida (Our Life)” i.e., as a reminder and also, to verify if it had influenced the subject in a positive way to perform daily routines as well as whether the influence had been diluted over the course of the days or not. Three subjects were excluded because of problems in filling out the TMRD by their parents. Therefore, the final sample used in the statistical analysis was composed of 27 subjects, 16 of whom were male and 11 females, all aged between 10 and 22 (mean age = 15.22; standard deviation = 2.88). A majority of these subjects are aged between 10 and 19. Additionally, 25 subjects already had experience with educational games.

The behavior of the subjects during the intervention phase with the virtual environment was observed and recorded by the researcher. For this purpose, a form (see Supplementary Material II) was used to collect the following data about their behavior during the interaction with the situations experienced in the virtual environment such as: presented difficulties to start the game, verbalized positively about the game, demonstrated interest in interactive activities, requested help, learned to use the controls easily, distracted themselves with external stimuli, requested extension of the pre-established time, played without stopping, and wanted to play again. The subjects were also questioned by the researcher if they owned a computer at home and were used to playing games to relate the recorded difficulties to the lack of motor coordination when using the mouse or keyboard.

### Experts evaluation and software tests

Software tests such as white box and black box were performed. To observe the quality of the implemented method, software tests were performed as required using capability maturity model integration (CMMI), ISO/IEC 9116 A, and ISO/IEC 14598 standards. For this, a test script that covers all the functionalities of all environments (playground, backyard, living room, bedroom, kitchen, and bathroom) was created to verify all sequences of actions and whether their respective feedback was occurring as expected. The logic pattern and system constraints were checked. The system has a standard of three logical choices where you can have one, two, or three correct choices. These tests were performed by the author.

An evaluation of eight specialists was also requested, of whom two were psychologists, three pedagogues, and three experts in games development. Their participation was limited to the execution of the virtual environment “Nossa Vida (Our Life)” v.2.0, for the date and place of their preference, taking into consideration the requirements implemented from the point of view of their expertise. To assist them in drafting the technical opinion, an evaluation form was presented to them by the research group. They could also report errors identified during the execution of the virtual environment such as difficulty in navigating or restarting an activity; and whether the visual and sound resources were pleasant and respond appropriately. Table 4 lists the profiles of professionals who voluntarily participated in the evaluation of the virtual environment “Nossa Vida (Our Life)”. They also signed the free and informed consent form.

**Table 4:** Professional Evaluators’ Profile.

Expert	Academic Education	Expertise
Psy1	Psychology	Acting in early childhood education
Psy2	Psychology	Acting in special education
Ped1	Pedagogy	Acting in technical and higher education
Ped2	Pedagogy	Kindergarten school owner
Ped3	Pedagogy	Teacher of early childhood education
GS1	Computer Science	Games specialist and Coordinator of Higher Education Course in Information Technology
GS2	Computer Science	Games specialist and Coordinator of the Digital Games Technology Course.
GS3	Computer Science	Games specialist and Teacher of Higher Education Course in Information Technology

The experts involved with our study have a theoretical background in psychology, psycho-pedagogy, pedagogy, information technology and game development.

The pedagogues verified the pedagogical requirements. If the educational content implemented in the virtual environment really bring benefits to the users’ learning; If the linguistic abilities are considered according to the source established by the Ministry of Education and Culture (MEC) for the rooms of special education; if the playful characteristics implemented can stimulate the logical reasoning of children with Down syndrome and can stimulate the memorization of daily routines and their creativity.

Psychologists, in turn, have observed the existence of rules and limits in the game, if it puts players in the face of winning or losing situations. They also checked whether the frustration of losing has been worked out in a positive way; if the functional requirements were implemented taking into account the characteristics of children with DS and can assist them in memorizing daily routines.

Games development experts have observed the following features: functionality, gameplay, usability and similarity of the graphical interface of the “Our Life” environment with the interface of commercial entertainment games.

### Statistical analysis

Statistical analysis was performed with software Bioestat 5.3. Homogeneity of the sample was verified using the Mann–Whitney test (α = 0.05), whereas the normality in the group was verified with D’Agostino’s test (α = 0.05). The existence of significant difference between the subjects’ performance in each group, namely control (GC) and experimental (GE), before (pre-test) and after (pos-test) the intervention performed with the virtual environment “Nossa Vida (Our Life)”, was verified with paired t’student test (α = 0.05). The existence of significant difference between the groups GC (before and after) versus GE (before and after) were verified using the Kruskal–Wallis test (α = 0.05).

Two hypotheses were tested in this study: H0 and H1, where: H0 = There is no statistical difference between the memorization of daily tasks between subjects with Down Syndrome who used our ludic virtual environment and those who used the conventional method of memory. H1 = There is a difference between the group of subjects with Down Syndrome who used our virtual play environment and the group that did not use in relation to the memorization of the daily task.

### Description of the virtual environment applied

The virtual environment applied in this study, named “Nossa Vida (Our Life) v.1.0”, was developed after interactions with a multidisciplinary team composed of nursery teachers (pedagogues and psycho pedagogues) and teachers of psychology and computer engineering, as suggested in the literature^44,99^. The elicitation of the functional requirements was carried out to identify the needs of the professionals of APAEs (that was visited by author in the state of São Paulo, Brazil) and especially of the children themselves regarding the memorization of actions sequences, which compose their daily routines.

These APAE professionals apply Piaget’s theory. According to Mantoan^100^, this theory refers to an education with liberal and democratic principles, in which education is adapted to each individual, providing the student with the conditions to form their own knowledge, by making correlations between events and objects that they interact. Thus, in order to obtain constructive education for students with mental disabilities, it is necessary to consider their capacity for cognitive and affective construction, starting from their personal needs and resources. In addition, to retain the attention of students with Down syndrome, teachers make use of the playful and phonic method.

Therefore, to implement the first version of the virtual environment, some of the several daily routine tasks that could be performed by the children in their own home, such as in environments of the kitchen, bathroom, living room, bedroom, and backyard were selected. According to the multidisciplinary team of APAE, the virtual environment should introduce the children to a scenario that explores something which is familiar to them.

The chosen theme was “Our Life”, which has two brothers as its main characters namely, Ana and Pedro aged 8 and 9 years respectively, and who are spending the weekend at their residence. The mother acts as a non-player character in the game, informing the child about what she should do whenever three consecutive decisions regarding her daily routines have resulted in a wrong choice^101^. Moreover, for the introduction to the playing, which is the child’s natural way of apprehending the world around him, the virtual environment was designed to have a nice graphical interface; plot and interactivity mechanisms; i.e., characteristics that define the computerized play. According to the literature, play environments can favor motivation, desire to finish a task, creativity, knowledge exchange, and the sharing of plans and emotions with other children, thereby contributing to intellectual development^44,102,103^.

The virtual environment generates immediate rewards for users (children) creating more stimulus for success. In all environments (phases) sound effects were inserted with the purpose of stimulating and motivating the development or completion of the activities^104^, i.e., to motivate or stimulate the child to execute sequences of daily actions aiming at their memorization.

**Figure 6:**
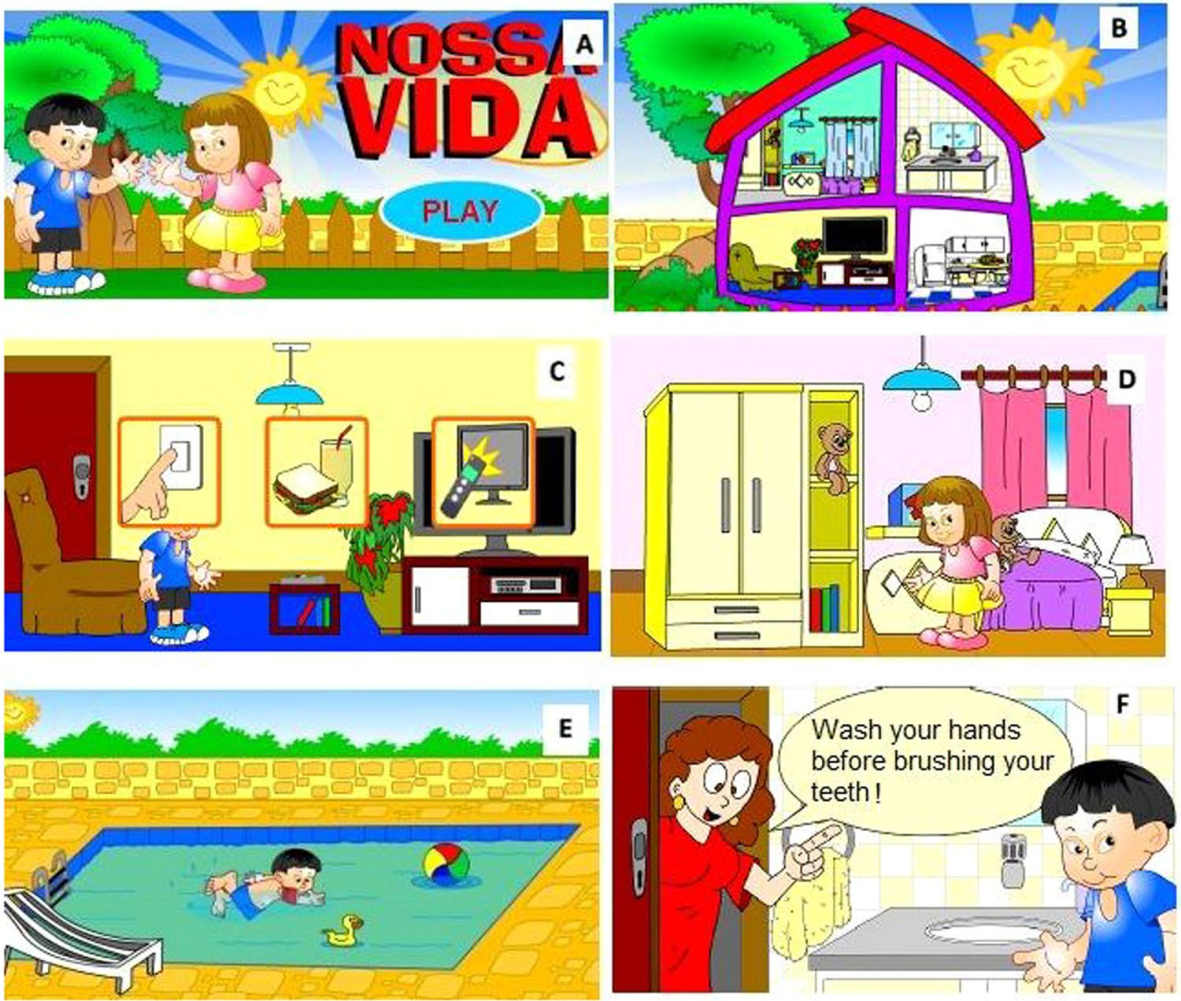
Some interfaces implemented in the “Nossa Vida (Our Life) 1.0”^45^ for child: (**A**) to start choosing the character with which the child wants to explore this virtual environment; (**B**) to choose the environments inside the house that were implemented; (**C**) to perform a decision making in the living room; (**D**) to stay at room environment; (**E**) to use swimming pool taking into account the good safety practices in the water; and (**F**) Interface where the mother appears providing a tip whenever the user makes three consecutive errors.

The subject should at first follow a logical sequence of actions normally performed by a child as a daily routine in his residence and then can make the transitions between the scenarios he wants through the main scenario represented by the interface which has the image of the house segmented in five environments. After that, the child can access the environments (phases) following an order of execution as many times as desired. The interaction of the child with the virtual environment is performed in first person through the use of the mouse, which is the form of control used in the games currently, and is also suitable for children with Down syndrome, according to APAE professionals. Figure 6 illustrates the user-friendly interfaces of some environments implemented in this first version of our software in accordance to the master’s thesis of the first author^63^.

To verify the consistency of this virtual environment, software tests (usability, navigability, and functionality) have been executed as well as evaluations of specialists in early childhood education (four pedagogues and three psychologists) and three specialists in educational software development have been carried out. These tests were considered necessary and sufficient for verifying if the functional and non-functional requirements as well as the pedagogical and psychological requirements were met.

## Supplementary information

Supplementary material.
