# Supplementary material for: Memorization of daily routines by children with Down syndrome assisted by a playful virtual environment
Source: Sci Rep. 2020 Feb 21;10:3144. doi: 10.1038/s41598-020-60014-5 (PMC7035352; doi:10.1038/s41598-020-60014-5)

|  |  |  |
| --- | --- | --- |

**MEMORIZATION OF DAILY ROUTINES BY CHILDREN WITH DOWN SYNDROME ASSISTED BY PLAYFUL VIRTUAL ENVIRONMENT**

Ovidio Lopes da Cruz Netto^b,d^, Silvia Cristina Martini Rodrigues^a,d^, Marcus Vasconcelos de Castro^b^, Diego Pereira da Silva^a,d^, Robson Rodrigues da Silva^a,c,d^, Richard Ribeiro Brancato de Souza^a,d,f^, Adriana A. Ferreira de Souza^g^, Marcia Aparecida Silva Bissaco^a,d,e*^

^a^ Technological Research Center, University of Mogi das Cruzes. Mogi das Cruzes, SP, Brazil.

^b^ Nove de Julho University (UNINOVE), São Paulo, SP, Brazil.

^c^ Center for Biomedical Engineering, University of Campinas, Campinas, SP, Brazil.

# ^d^ Postgraduate Programme in Biomedical Engineering, University of Mogi das Cruzes. Mogi das Cruzes, SP, Brazil.

# ^e^ Professional Master's program in Health Science and Technology, University of Mogi das Cruzes. Mogi das Cruzes, SP, Brazil.

^f^ Behavior Analysis Core, São Paulo, SP, Brazil.

^g^ Psychology Clinic, University of Mogi das Cruzes

**Corresponding author:**

Marcia Aparecida Silva Bissaco

University of Mogi das Cruzes, Technological Research Center.

Av. Dr. Cândido Xavier de Almeida, 200.

Mogi das Cruzes, SP, Brazil.

CEP: 08780-911.

Phone: +55 (11) 4798-7228. Fax: +55 (11) 4799-2069

E-mail: [marciab@umc.br](mailto:marciab@umc.br), mbissaco@gmail.com

OLCN: ovidio@uninove.br

SCM: silviac@umc.br

MVC: marcusdecastro@uninove.br

DPS: diego.silva@umc.br

RRS: robson.silva@umc.br

RRBS: r.brancato@hotmail.com

AAFS: [adrianaaf@umc.br](mailto:adrianaaf@umc.br)

MASB: [marciab@umc.br](mailto:marciab@umc.br), mbissaco@gmail.com

**Daily Routine Memorization Test (DRMT)**

An instrument for data collection developed by the authors with the aid of a psychologist. It is a weekly reminder of typical daily household routines to be completed by the children and their parents.

The frequency that the action was performed on each day of the week should be recorded on this form in the cell corresponding to that day.

# Child's name: _____________________________________________________

| **Sequence of Actions** | **Days of the week** | | | | | | |
| --- | --- | --- | --- | --- | --- | --- | --- |
|  | **Mon** | **Tue** | **Wed** | **Thu** | **Fri** | **Sat** | **Sun** |
| 1) When you enter the bathroom, turn on the light |  |  |  |  |  |  |  |
| 2) When brushing your teeth, wash your hands before. |  |  |  |  |  |  |  |
| 3) At the end of brushing your teeth, dry yourself with the towel. |  |  |  |  |  |  |  |
| 4) Before you leave the bathroom turn off the light. |  |  |  |  |  |  |  |
| 5) Enter the kitchen to eat |  |  |  |  |  |  |  |
| 6) When eating an apple, wash it first. |  |  |  |  |  |  |  |
| 7) When you finish eating an apple, throw the leftovers in the trash. |  |  |  |  |  |  |  |
| 8) When eating a banana, peel it before. |  |  |  |  |  |  |  |
| 9) When you finish eating a banana, throw the peel in the trash. |  |  |  |  |  |  |  |
| 10) Before leaving the kitchen, turn off the light. |  |  |  |  |  |  |  |
| 11) When you enter the bedroom, turn on the light. |  |  |  |  |  |  |  |
| 12) When going to sleep, put on your pajamas before. |  |  |  |  |  |  |  |
| 13) When you get your bed to sleep, take away everything that is on it before. |  |  |  |  |  |  |  |
| 14) When playing with a puzzle, make more than one puzzle. |  |  |  |  |  |  |  |
| 15) Before leaving the bedroom, turn off the light. |  |  |  |  |  |  |  |
| 16) When swimming in a pool, place the buoys first. |  |  |  |  |  |  |  |
| 17) When you are swimming, eat something |  |  |  |  |  |  |  |
| 18) Usually swim for a long time. |  |  |  |  |  |  |  |
| 19) Usually play with objects while swimming. |  |  |  |  |  |  |  |
| 20) When you leave the pool, the first thing you do is take out the buoys. |  |  |  |  |  |  |  |
| 21) When you enter the living room, turn on the light. |  |  |  |  |  |  |  |
| 22) When watching TV, keep pressing the buttons on the remote control before turning on. |  |  |  |  |  |  |  |
| 23) When you take the remote control of the TV, you already press the button to turn it on. |  |  |  |  |  |  |  |
| 24) Usually play with objects while swimming. |  |  |  |  |  |  |  |
| 25) Turn the TV on and off constantly. |  |  |  |  |  |  |  |
| 26) Change the TV volume frequently. |  |  |  |  |  |  |  |
| 27) Before leaving the living room, turn off the light. |  |  |  |  |  |  |  |

# Additional comments: ______________________________________________________________________

# ______________________________________________________________________

# ______________________________________________________________________

# Period: ___ / ___ / ___ to ___ / ___ / ___

# Responsible: __________________________________________________________

#

# **Form for recording children's behavior**

# Child's name: _____________________________________________________

# Do you have a computer at home? (YES NO)

# Do you play on your computer? (YES NO)

# Instrument for collecting behaviors inspired by Castro et al (2014)

| Behaviors | **Number of children** |
| --- | --- |
| They had difficulties to start the game |  |
| They asked for help |  |
| They distracted themselves with external stimuli |  |
| They asked for an increase in the pre-established time period |  |
| Played nonstop |  |
| Positive verbal about the game |  |
| Learned to use controls easily |  |
| They wanted to play again |  |

**Additional Material about the virtual environment developed “Nossa Vida (Our Life)”**

1. **Complete description of the diagrams of the cases of use**

Figure 1: Case of use 001- Starting options contemplating the customization of the character and the photo taking as well as the access to the recreation.

| **Case of use UC001: Starting Options** |
| --- |
| **Description:** This case of use allows the user to select a functionality that wants to execute in the beginning of the system.  **Precondition:** The user must have a WIFI connection and must be logged in.  **Main Flow:**   1. The system exhibits a starting message. 2. The system exhibits the main screen. 3. The user selects the character. 4. The system shows a button to start. 5. The user clicks on start 6. The system redirects to the main screen.   **Alternative Flow:**  **A1: Select photo**  The sequence A1 starts from point 3 of the main flow.   1. The user selects the photo option. 2. The system loads screen of personification of the ambience and character to make the photo.   **A2: Select background**  The sequence A2 starts from point 3 of the main flow.   1. The user select the option playground 2. The system loads the playground screen |

Figure 2: Case of use 002 – Visualize reports

| **Case of use UC002: Visualize reports** |
| --- |
| **Description:** This case of use allows the user to be authenticated in the web server through the name of the user, the password and visualize reports of game usage.  **Precondition:** The access to the other system screens will only be permitted to users that are registered in the web server.  **Main Flow:**   1. The system asks for user and password. 2. The user puts the user and password. 3. The system validates the data comparing with the information registered on the banco of data by using the web server. 4. The system shows a message informing that the authentication was executed with success. 5. The system redirects the user to the report viewing screen.   **Alternative Flow:**  **A1: user name nonexistent**  The sequence A1 starts from point 3 of the main flow.   1. The system shows a message informing that the user and password are wrong. |

Figure 3: Case of use 003- Select photo

| **Case of use UC003: Select photo** |
| --- |
| **Description:** This case of use allows the user to personalize their character and save a picture.  **Precondition:** The user must have selected the photo option in the use of case UC001 beginning options.  **Main Flow:**   1. The system loads a screen 2. The user select a character 3. The user selects the character’s hat 4. The user selects the character’s shirt. 5. The user selects the character’s pants.   **Alternative Flow:**  **A1: Save a picture**  The sequence A1 starts from point 1 of the main flow.   1. The user can save a Picture of the character’s personalization.   **A2: Change scenario**  The sequence A2 starts from point 1 of the main flow.   1. The user can change the scenario |

Figure 4: Case of use 004- Select Playground

| **Case of use UC004: Play in the playground** |
| --- |
| **Description:** This case of use allows the user to interact with the various items that exist in the playground.  **Precondition:** The user must have selected a playground option in the UC001 case of use.  **Main Flow:**   1. The user drags the character to the toy. 2. The user drags the second character to the toy. 3. The system performs the action to play on the toy   **Alternative Flow:**  **A1: user name non-existent**  The sequence A1 starts from point 3 of the main flow.   1. The system shows a message informing that the user and password are wrong. |

Figure 5: Case of use 005- Living Room

| **Case of Use UC005: Living Room** |
| --- |
| **Description:** This case of use allows the user to interact with the items that exist in the room.  **Precondition:** The user must have selected the room option in the case of use UC001 Initial Options.  **Main Flow:**  1. The system shows a message that the remote control is on the table.  2. The system displays the options.  3. The user selects an option.  4. The system validates if the selected option was to turn on the lights  5. The system changes the ambient light.  6. The system displays the options.  7. The user selects an option  8. The system validates if the selected option was to pick up the control.  9. The system redirects the user to the television screen.  10. The system shows a message with instructions.  11. The system displays the options.  12. The user selects an option.  13. The system validates if the option was to watch tv.  14. The system loads the channels.  **Alternative Flow:**  **A1: Turn on the lights**  The sequence A1 starts from point 4 of the main flow.   1. The system shows a review indicating that the selection is wrong. 2. **A2: Eat lunch**   The sequence A2 starts from point 4 of the main flow.  1. The user selects the eat snack option.  2. The character issues a critique indicating that the selection is wrong.  **A3: Change the volume**  The sequence A3 starts from point 14 of the main flow.   1. The user can select options to increase or decrease the volume. 2. The user can select the mute option 3. **A4: Change the channel**   The sequence A4 starts from point 14 of the main flow.   1. The user can select channel switch options. 2. The user can select the TV on / off option. |

Figure 6: Case of use 006- Kitchen

| **Case of use UC006: Kitchen** |
| --- |
| **Description:** This case of use allows the user to interact with some items that exist in the kitchen.  **Precondition:** The user must have selected the kitchen option in the UC001 Initial case of use.  **Main Flow:**   1. The system issues a message stating instructions for the user to make the character eat something and throw the remains in the trash. 2. The system displays the options. 3. The user selects an option. 4. The system validates if the selected option was to turn on the lights 5. The system changes the ambient light. 6. The system displays the options. 7. The user selects an option 8. The system validates if the selected option was to pick up the apple. 9. The system walks with the character and grabs the apple. 10. The system displays the options. 11. The user selects an option. 12. The system validates if the option was to wash apple. 13. The system displays the apple wash routine. 14. The system displays the options. 15. The user selects an option. 16. The system validates if the selected option was to eat the apple. 17. The character eats the apple. 18. The system displays the options. 19. The user selects an option. 20. The system validates if the selected option was to throw rest in the trash. 21. The character walks to the bin and discards the rest of the apple.   **Alternative Flow:**  **A1: Turn on the lights**  The sequence A1 starts from point 4 of the main flow.   1. The system shows a review indicating that the selection is wrong.   **A2: Select Banana**  The sequence A2 starts from point 9 of the main flow.   1. The user selects the banana option. 2. The system walked with the character and picks up a banana. 3. The system displayed as options. 4. The user selects an option. 5. The system validates if the option selected was to peel a banana. 6. The character peels a banana. 7. The system displayed as options. 8. The user selects an option. 9. The valid system is a separate option for eating banana. 10. The character sees a banana 11. The system displayed as options 12. The user selects an option 13. The valid system is not disposable 14. The character walks to the bin and discards the shell of the banana   **A3: Eat lunch**  The sequence A3 starts from point 4 of the main flow.   1. The user selects the snack option 2. The system walks with the character, opens the refrigerator and picks up the snack. 3. The system displays the options. 4. The user selects an option. 5. The system validates if the selected option was to open. 6. The character opens the pack of the snack. 7. The system displays the options. 8. The user selects an option. 9. The system validates if the selected option was to eat snack. 10. The character eats the snack 11. The system displays the options 12. The user selects an option 13. The system validates if the selected option was to throw the packaging in the trash 14. The character walks to the bin and discards the lunch pack |

Figure 7: Case of use 007- Bedroom

| **Case of use UC007: Bedroom** |
| --- |
| **Description:** This case of use allows the user to interact with the various items that exist in the room.  **Precondition:** The user must have selected the fourth option in the UC001 case of use Initial Options.  **Main Flow:**   1. The system shows a message stating instructions. 2. The system displays the options. 3. The user selects an option. 4. The system validates if the selected option was to turn on the lights 5. The system changes the ambient light. 6. The system displays the options. 7. The user selects an option. 8. The system validates if the selected option was to change clothes. 9. The character walks to the wardrobe. 10. The system displays the options. 11. The user selects an option. 12. 12. The system validates if the selected option was to put on pajamas. 13. The character changes clothes. 14. The system displays the options. 15. The user selects an option. 16. The system validates if the selected option was to put the toy away. 17. The character puts the toy away. 18. The system displays the options. 19. The user selects an option. 20. The system validates if the selected option was to sleep 21. The system changes the color of the environment 22. The character lies in bed and sleeps 23. The system displays the options 24. The user selects an option 25. The system validates if the selected option was to wake up 26. The system changes the color of the environment 27. The character wakes up. 28. The character changes clothes. 29. The system shows a message. 30. The system redirects uc001 Initial Options to the Usage Case.\   **A1: Turn on the lights**  The sequence A1 starts from point 4 of the main flow.   1. The system issues a review stating that it is necessary to turn on the lights   **A2: Sleep without changing clothes**  The sequence A2 starts from point 8 of the main flow.   1. The system issues a review stating that it is necessary to change clothes before bed   **A3: Select inappropriate clothing**  The sequence A3 starts from point 12 of the main flow.   1. The system issues a critique stating that it is necessary to wear pyjamas   **A4: Sleeping without putting the toy away.**  The sequence A4 starts from point 16 of the main flow.   1. The system issues a review stating that it is necessary to put the toy away before bed   **A5: Play while you sleep**  The sequence A5 starts from point 25 of the main flow.   1. The system issues a review stating that it is necessary to wake up before playing |

Figure 8: Case of use 008- Bathroom

| **Case of use UC008: Bathroom** |
| --- |
| **Description:** This use case allows the user to interact with the various items that exist in the bathroom.  **Precondition:** The user must have selected the bathroom option in the case of use UC001 Initial Options.  **Main Flow:**   1. The system shows a message stating the instructions. 2. The system displays the options. 3. The user selects an option. 4. The system validates if the selected option was to turn on the lights 5. The system changes the ambient light. 6. The system displays the options. 7. The user selects an option 8. The system validates if the selected option was to wash hands. 9. The character washes his hands. 10. The system displays the options. 11. The user selects an option. 12. The system validates if the option was to brush teeth. 13. The character brushes the teeth. 14. The system displays the options. 15. The user selects an option. 16. The system validates if the selected option was to dry your hands. 17. The character dries his hands   **Alternative Flow:**  **A1: Turn on the lights**  The sequence A1 starts from point 4 of the main flow.   1. The system shows a review indicating that the selection is wrong.   **A2: Brushing your teeth without washing your hands**  The sequence A2 starts from point 8 of the main flow.   1. The system displays a review stating that you should wash your hands first.   **A3: Wash hands again**  The sequence A3 starts from point 12 of the main flow.   1. The system shows a review indicating that the selection is wrong. |

Figure 9: Case of use 009- Pool

| **Case of use UC009: Pool** |
| --- |
| **Description:** This case of use allows the user to play with the selected character in the pool.  **Precondition:** There is not.  **Main Flow:**   1. The system sends the initial message requesting that the character does not forget to put the float. 2. The system displays the options. 3. The user selects an option. 4. The system validates if the selected option was the float. 5. The character wears the float. 6. The system displays the options. 7. The user selects an option. 8. The system validates if the selected option was to swim. 9. The character begins to swim 10. The system displays the options 11. The user selects an option. 12. The system validates if the selected option was to exit the pool 13. The character leaves the pool 14. The character removes the float   **Alternative Flow:**  **A1: Select swim**  The sequence A1 starts from point 4 of the main flow.   1. The character issues a critique indicating that the selection is wrong.   **A2: Select lunch**  The sequence A2 starts from point 8 of the main flow.   1. The character issues a critique indicating that the selection is wrong.   **A3: Select**  The sequence A3 starts from point 9 of the main flow.   1. The user selects the Menu option. 2. The system redirects the user to the UC001 case of use Initial Options. |

**2. INTERFACES OF THE VIRTUAL ENVIRONMENT “NOSSA VIDA (OUR LIFE) 2.0”**

This topic presents the main functionalities of the software, detailing the actions and answers, and showing the new illustrations. To detail the features, the female character will be used. The logic is identical for the male character. When starting the game the opening screen is presented according to Figure 10.

Figure 10: Game Opening Interface.


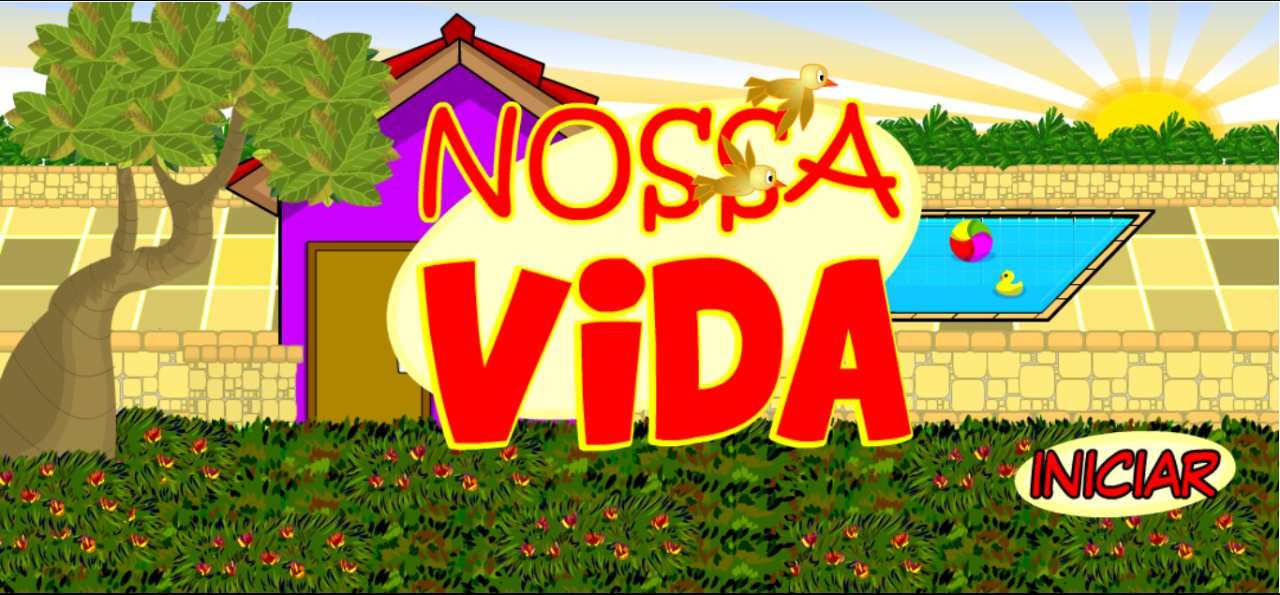


The text is presented with the verbal reading of it, then the child can choose the character if it is female or male and still customize it (Figure 11), choosing their clothes, accessories, environments or even take photos of their visual, obtaining the photo immediately through a PNG file instantly, which can be saved in place and with a name chosen by the user.

Figure 11: In this new version the game allows customization of the character and the environment.
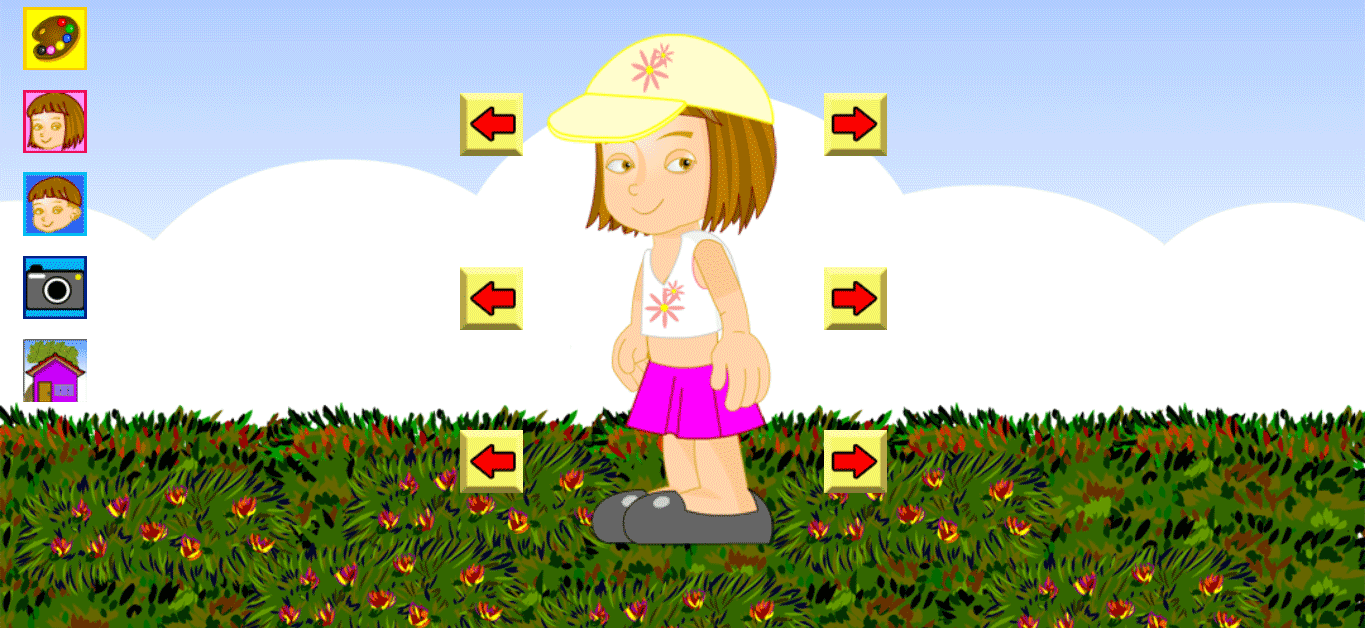


After the character has been personalized, the initial navigation interface is displayed, in which the environments that the child wishes to explore are chosen (Figure 12). Each interface and the logical actions that can occur in the interface are presented below.

- **Routine of the Initial Interface (for selection of environments to be explored)**

In the Interface shown in Figure 12 the child can choose, when running the free mode, the environment that wants to explore. If it is in the fixed sequence mode, it must begin its exploration by the environments that are being enabled.

**Figure 12:** Interface in which the child will choose the initial activities to be developed
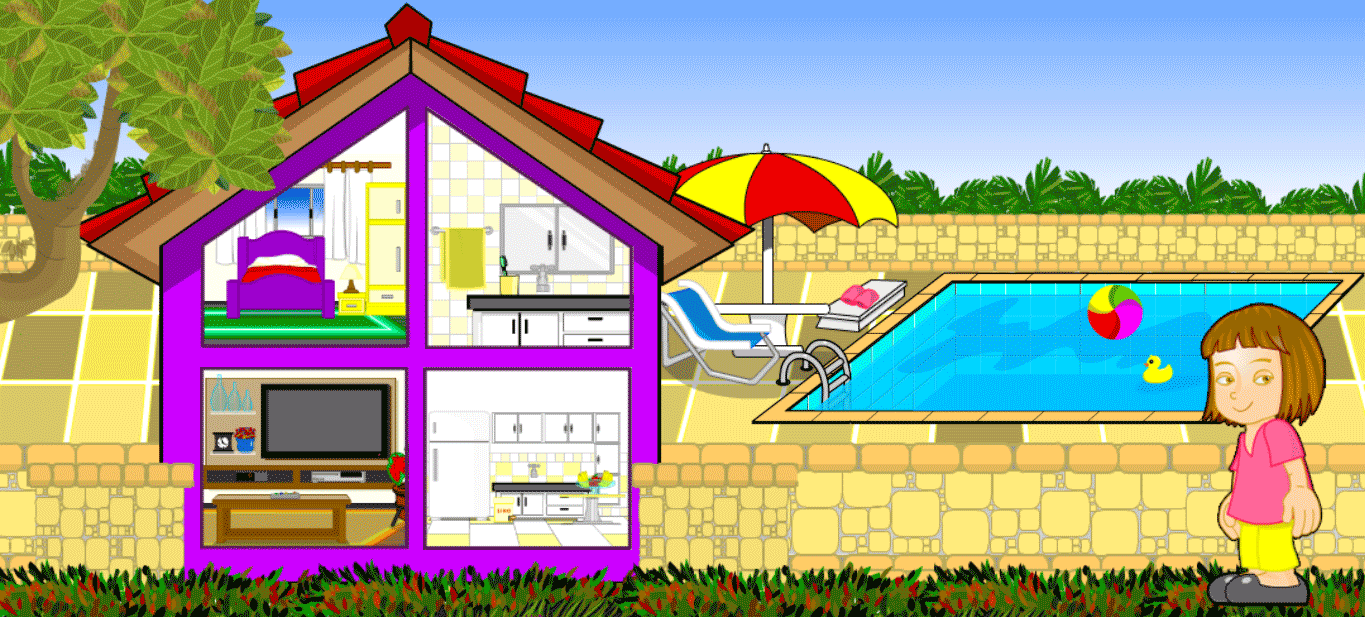
.

**• Room Routine**

When choosing the ROOM environment, the child can play with the puzzle and do the whole sleeping routine, which involves arranging the room by keeping the stuffed animal in the wardrobe and putting on a suitable bedding, and always at the beginning of the environment the mother appears giving directions of the sequences that will be performed in that environment as shown in Figures 13 to 19.

Figure 13: Interface of the Room with the instructions of the sequences that will be performed.


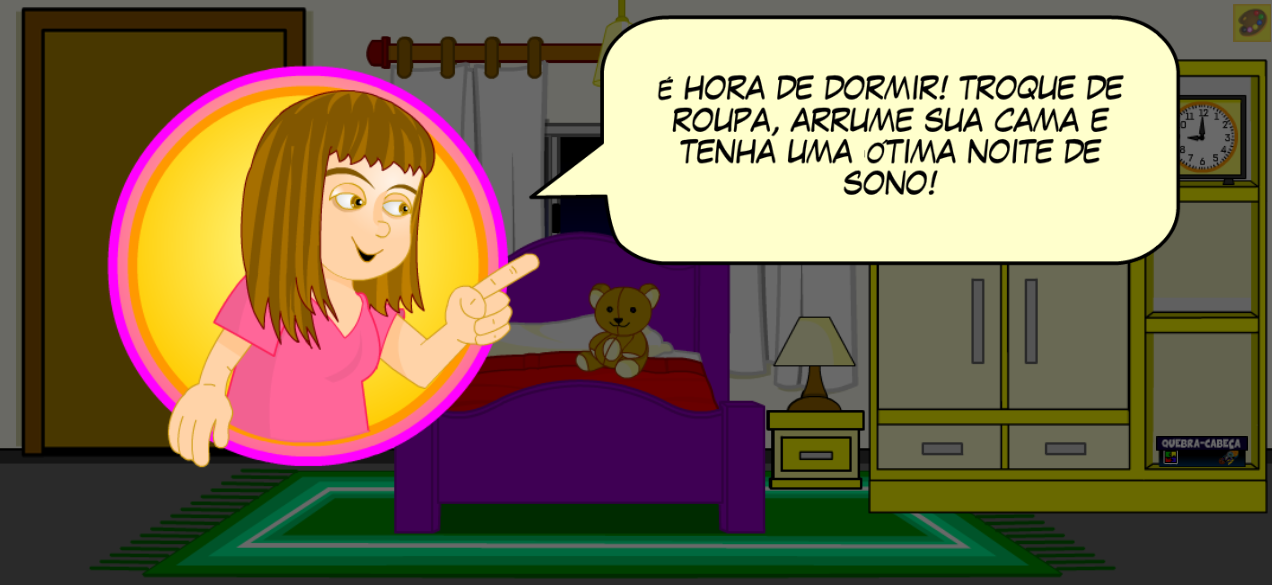


It’s time to sleep! Change your clothes, organize your bed and have a good night sleep!

Figure 14: Interface of the bedroom scenario where the child can choose which interaction will perform first.
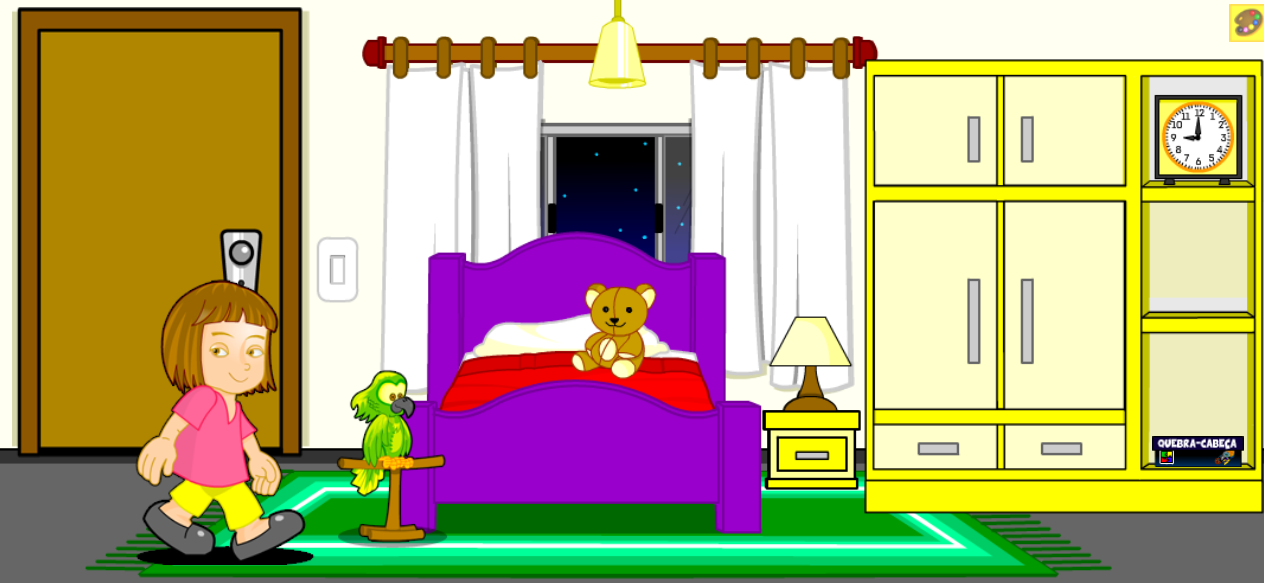


Figure 15: Interface of the bedroom scenario exposing the open wardrobe where the child can choose some clothing.


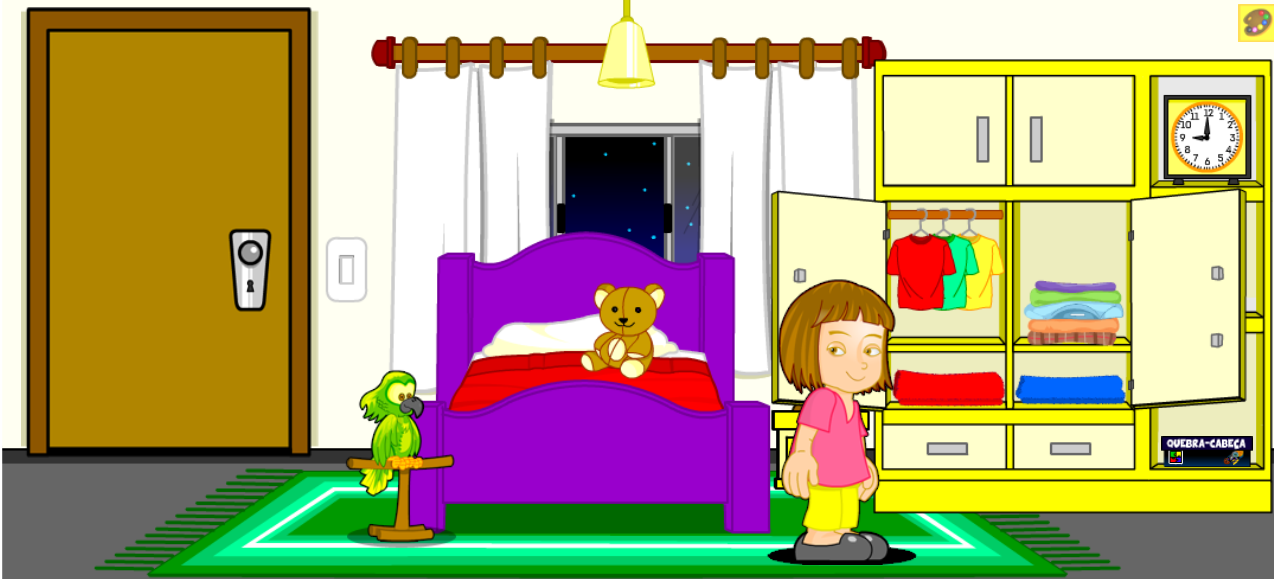


Figure 16: Interface that shows the child changing clothes.


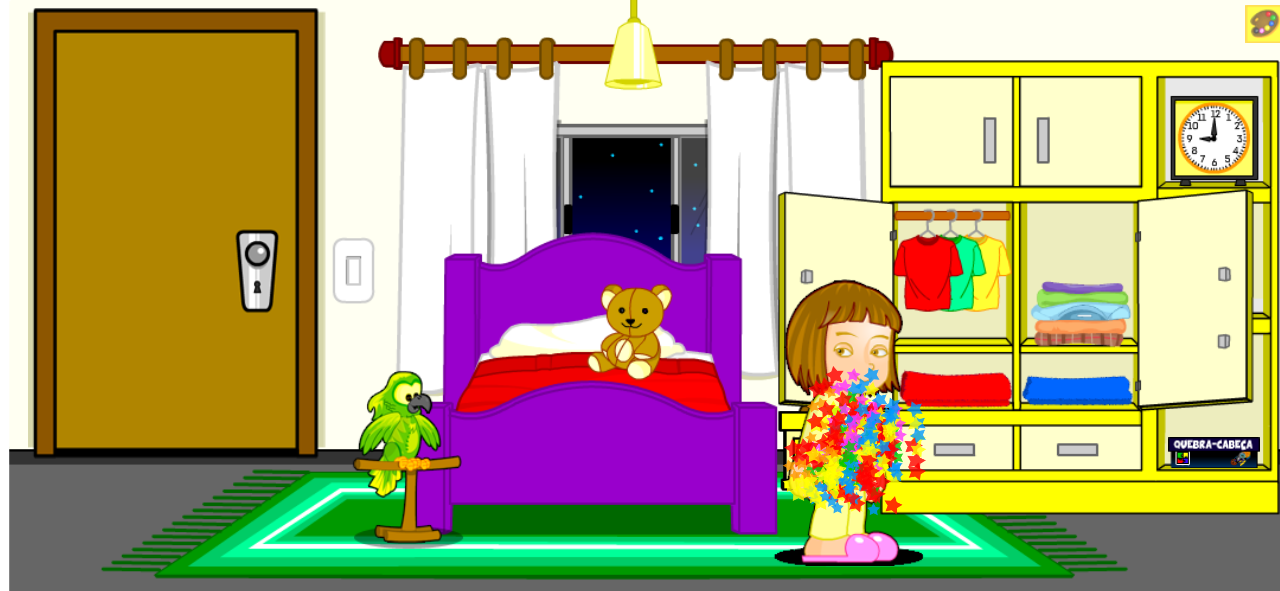


Figure 17: Room screen showing the child in pajamas putting the bear in the closet.


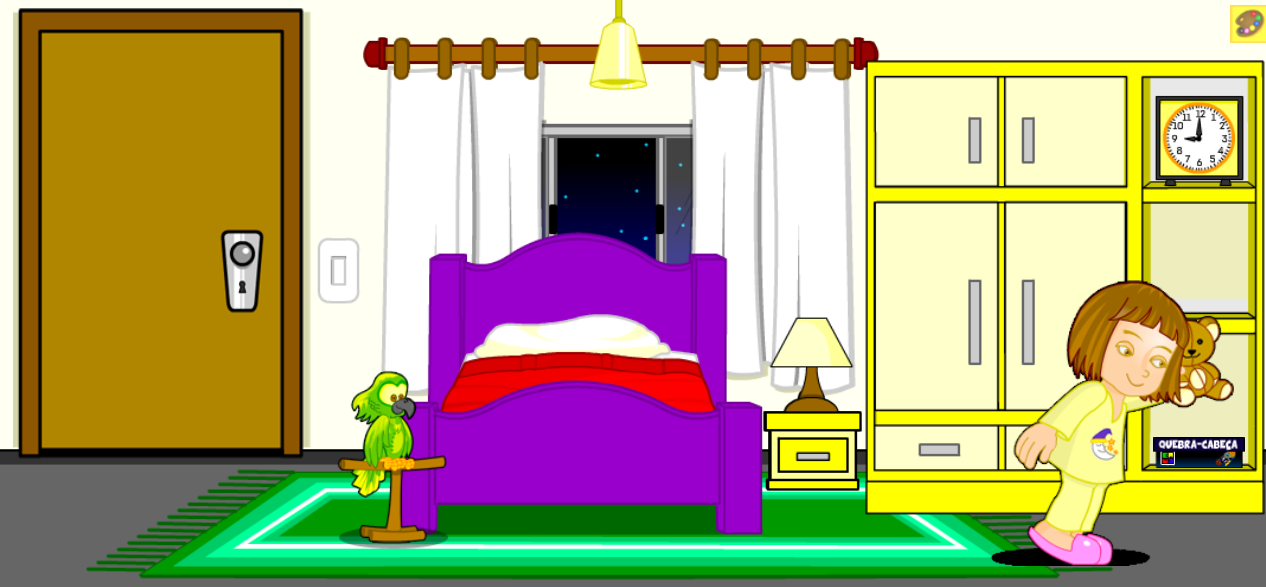


Figure 18: Screen showing the child sleeping


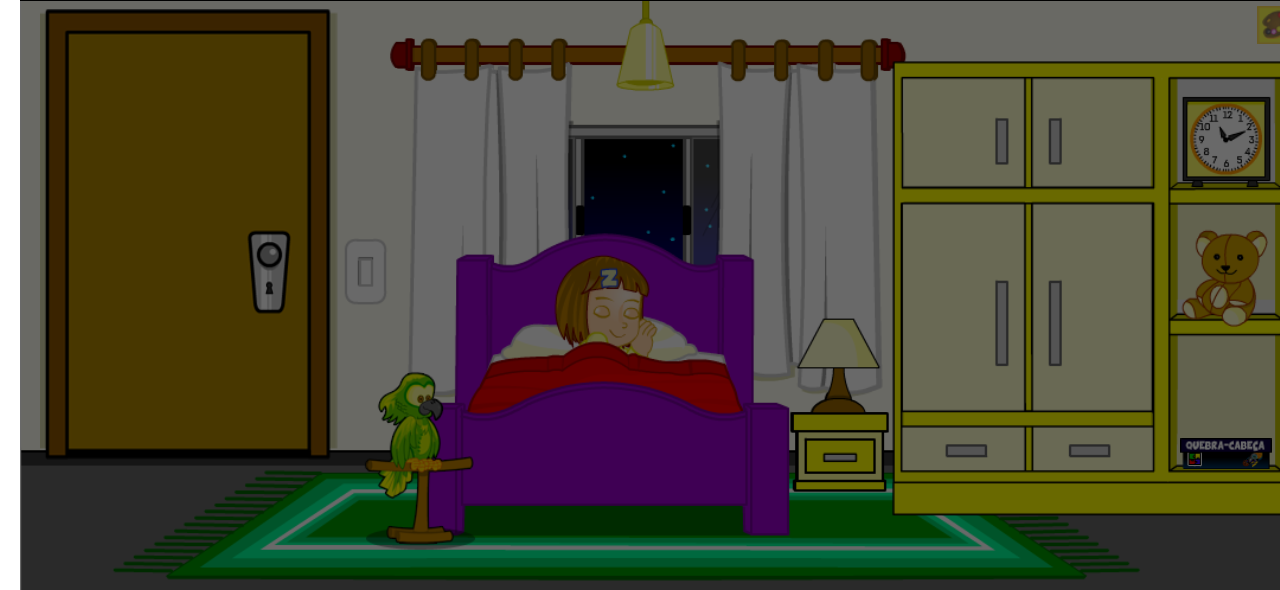


Figure 19: Mother feedback screen.


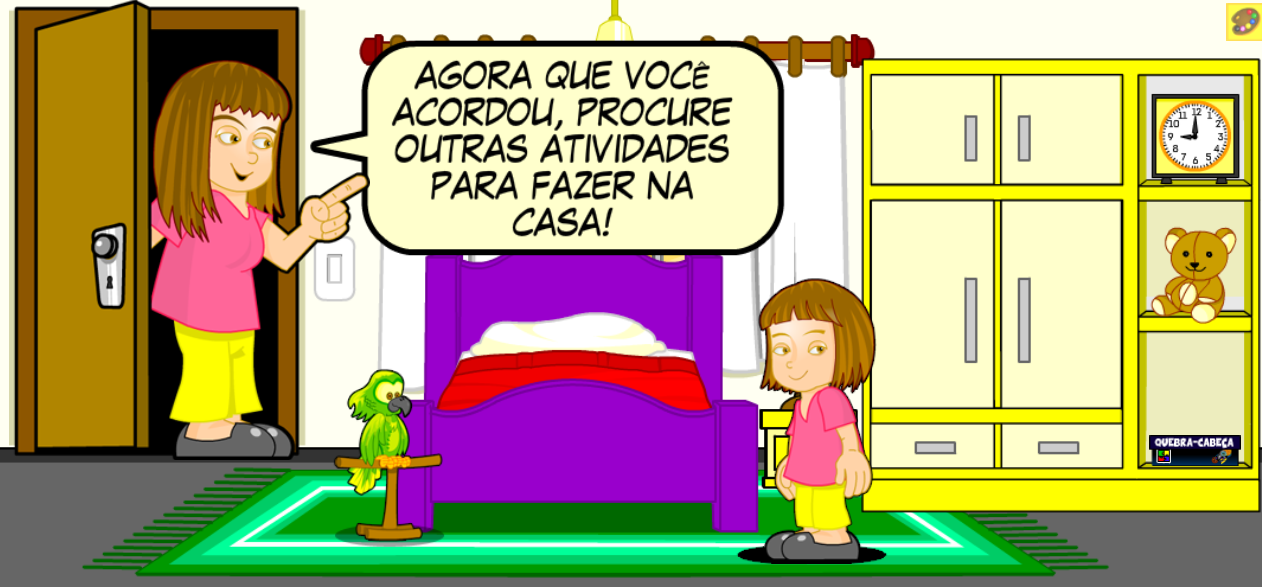


Now that you woke up, find other activities to do in the house!

• PLAYGROUND ROUTINE

In the PLAYGROUND, the children can play on the swing, seesaw and slide by simply dragging the character to the toys. To do this, before the user starts, the software presents a demonstration of how the user should control the characters, as shown in figures 20 to 23.

Figure 20: Playground screen


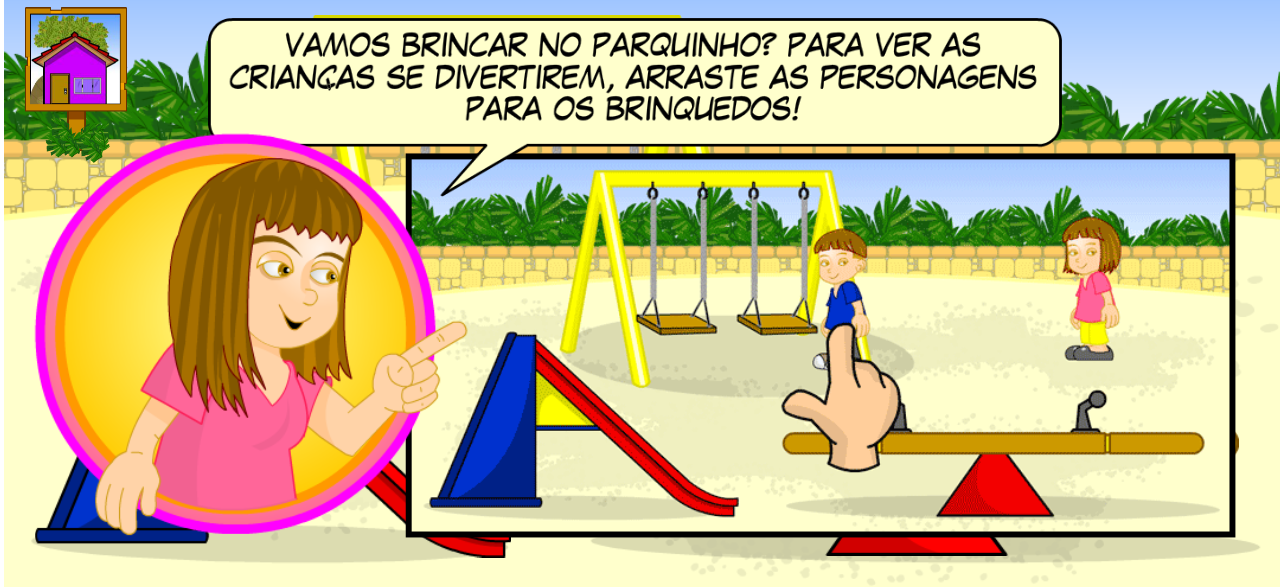


Let’s play in the playground? To see the children having fun, drag the characters to the toys!

Figure 21: Screen with children on the swing.


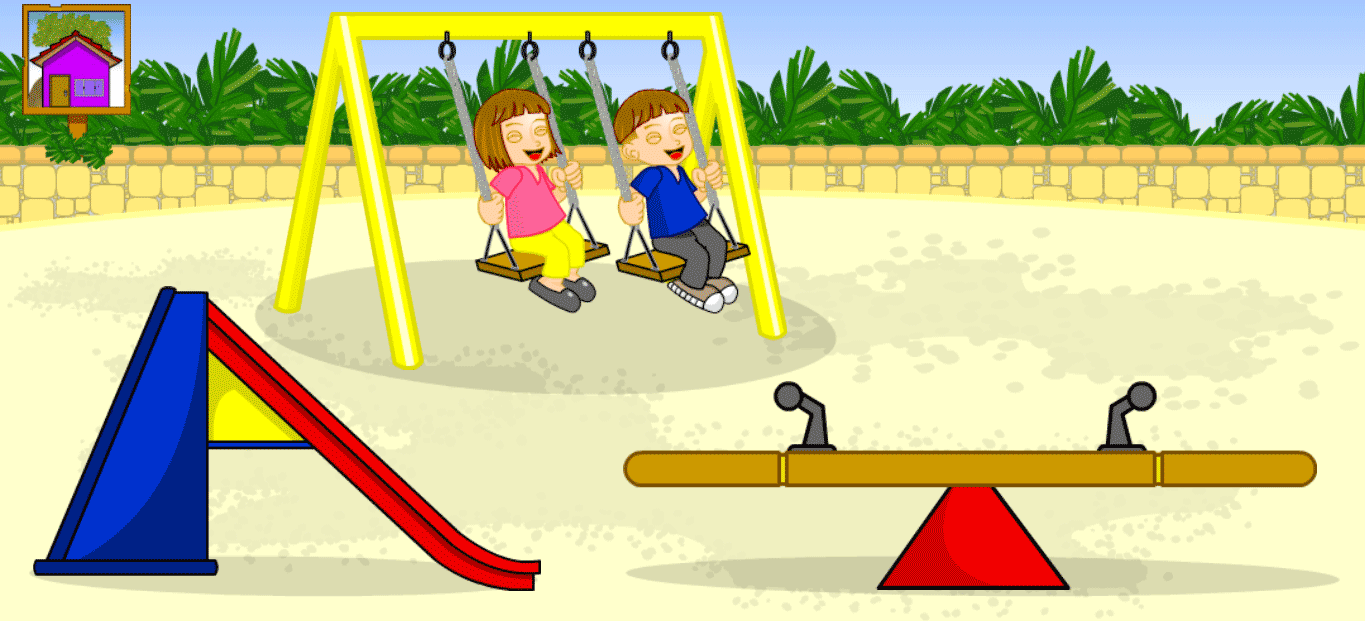


Figure 22: Playground screen with child using the slide.


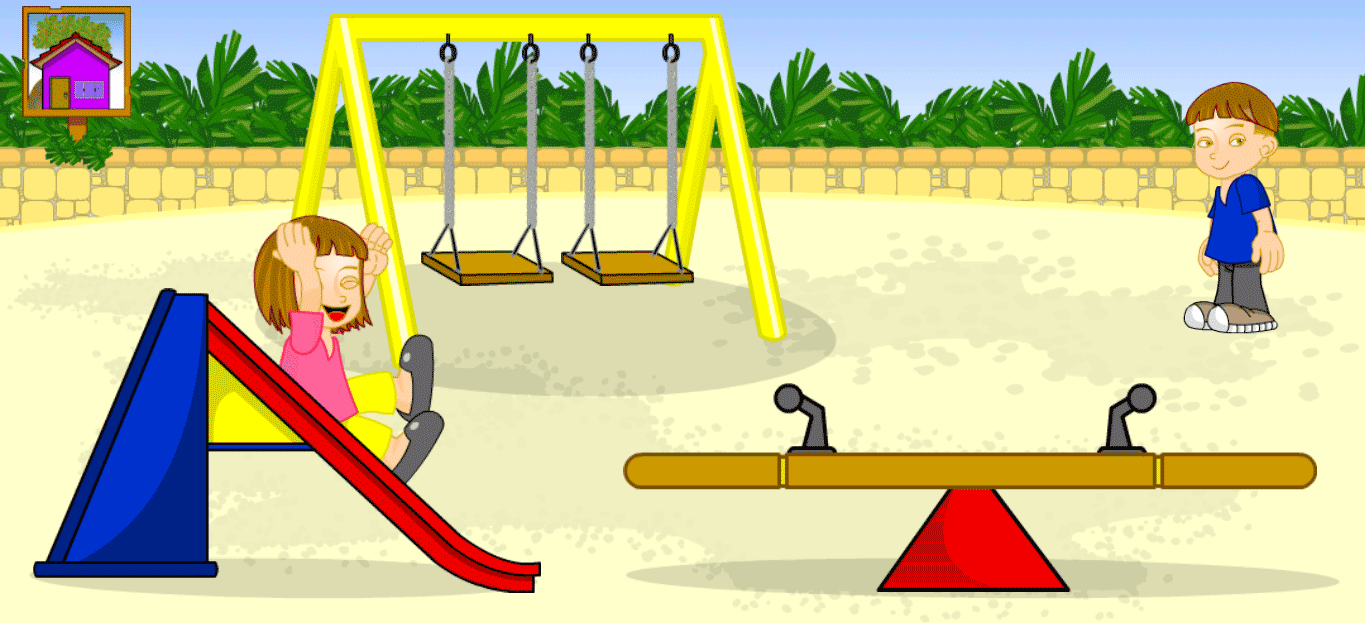


Figure 23: Playground screen with children on the seesaw.


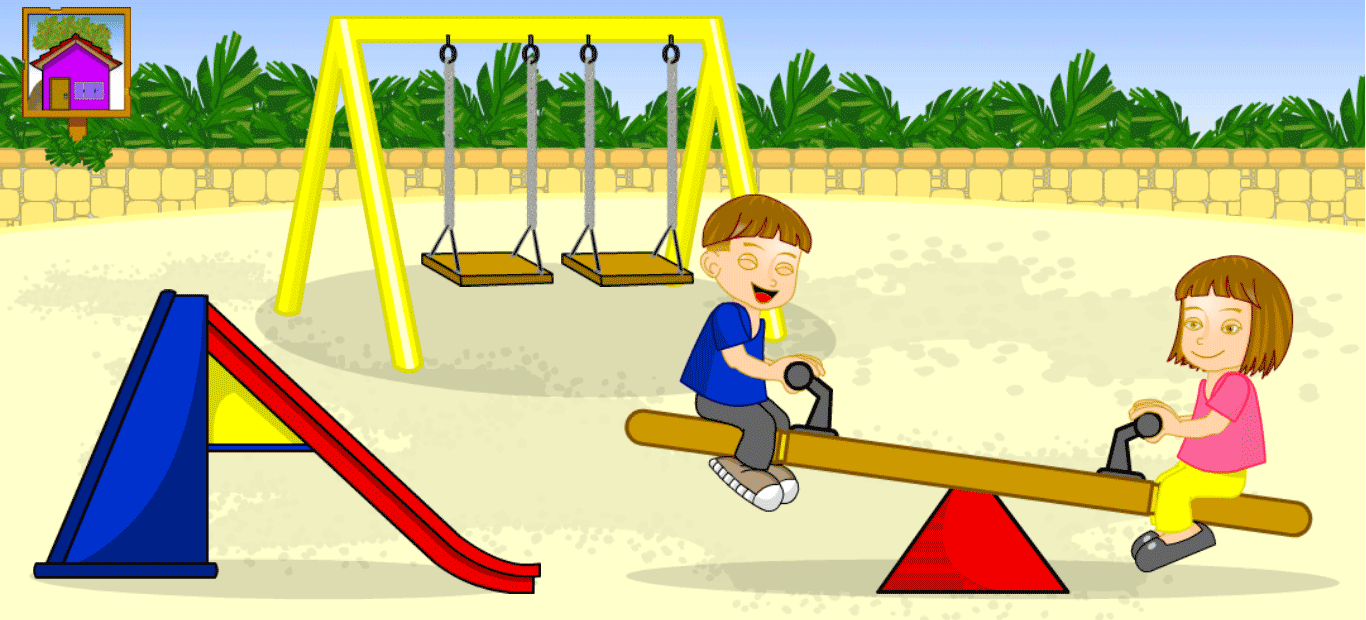


- **KITCHEN ROUTINE**

In the KITCHEN environment, the child can eat a fruit (apple and / or banana) and / or with a snack. However, for this you should perform the appropriate hygiene actions with all foods. In addition, at the beginning, a message from the mother will be displayed informing what can be done in this environment, as shown in the images in Figures 24 to 34.

Figure 24: Mother’s message stating what should be done in the kitchen.


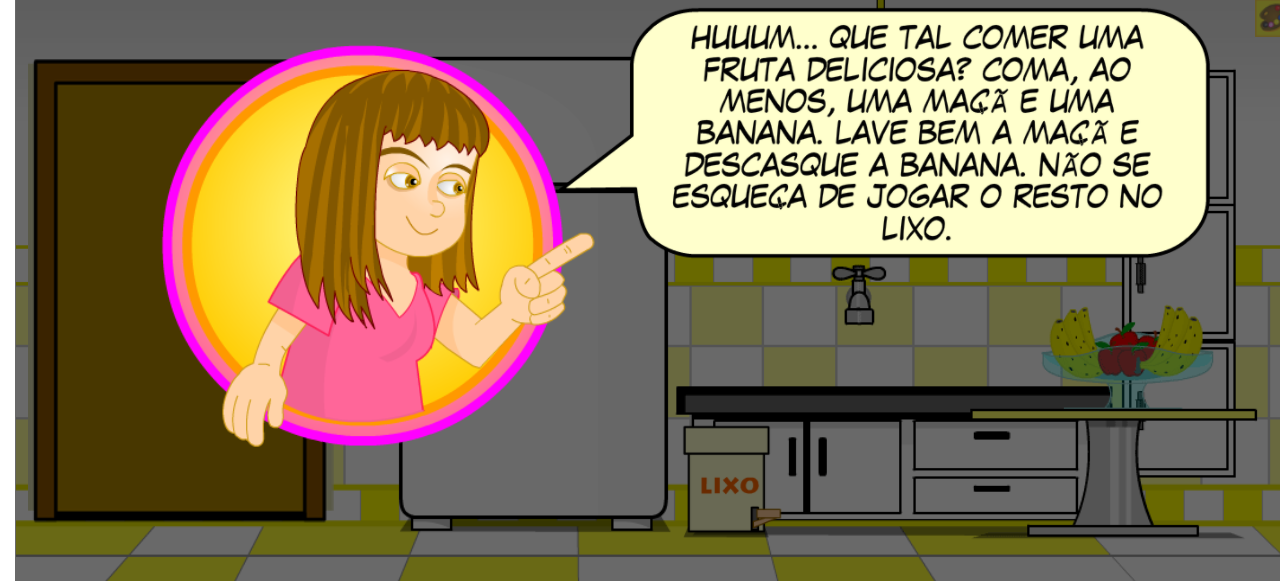


Hmmm….. How about eating an delicious fruit? Eat, at least, a apple and a banana. Wash the apple well and peel the banana. Don’t forget to put the rest in the trash.

Figure 25: Kitchen screen without light


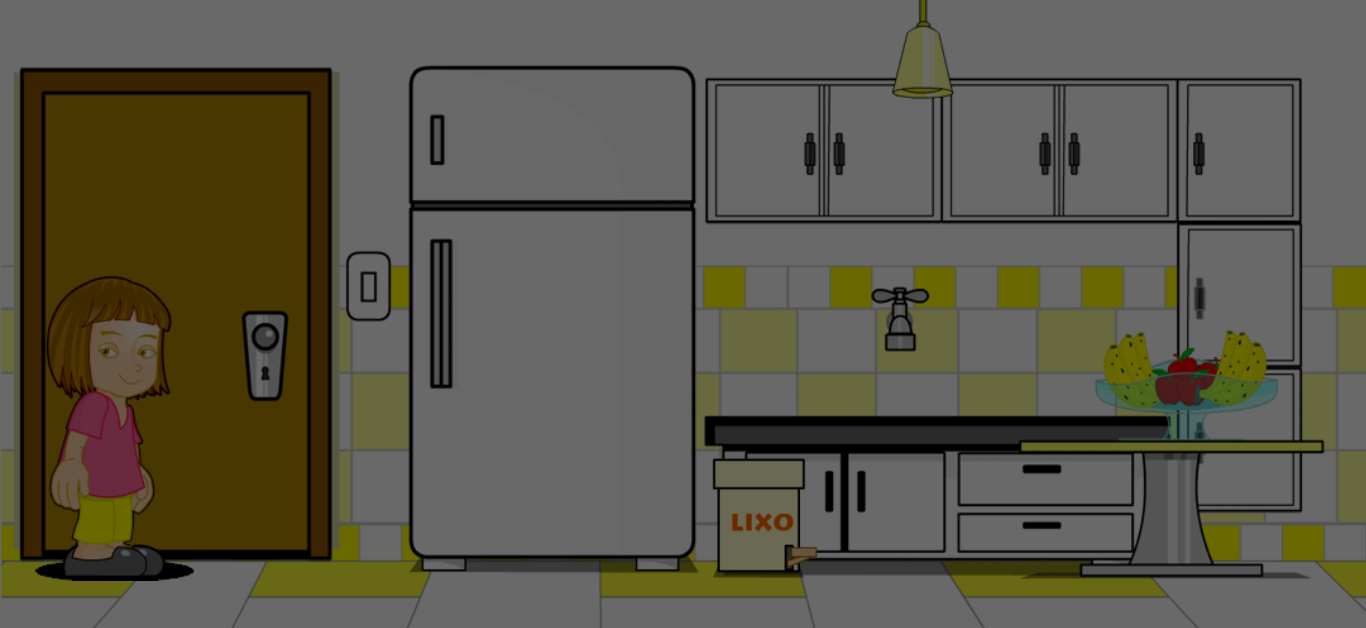


Figure 426: Kitchen screen with light


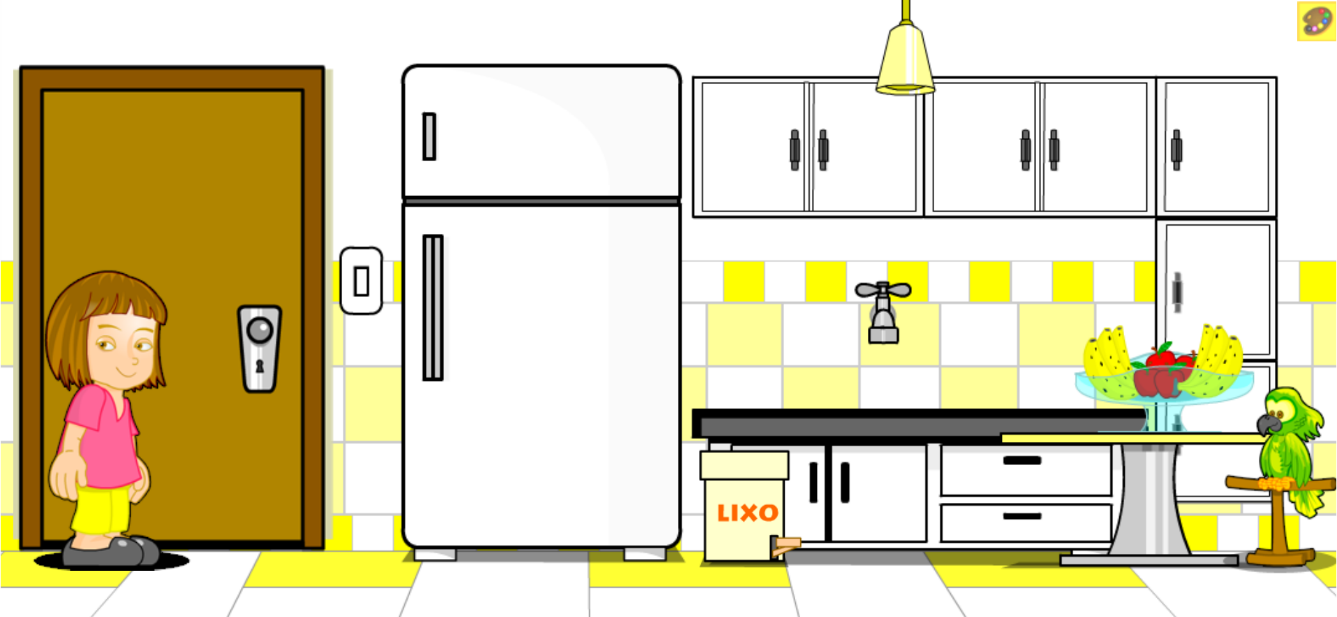


Figure 27: Kitchen screen with a child holding an apple.


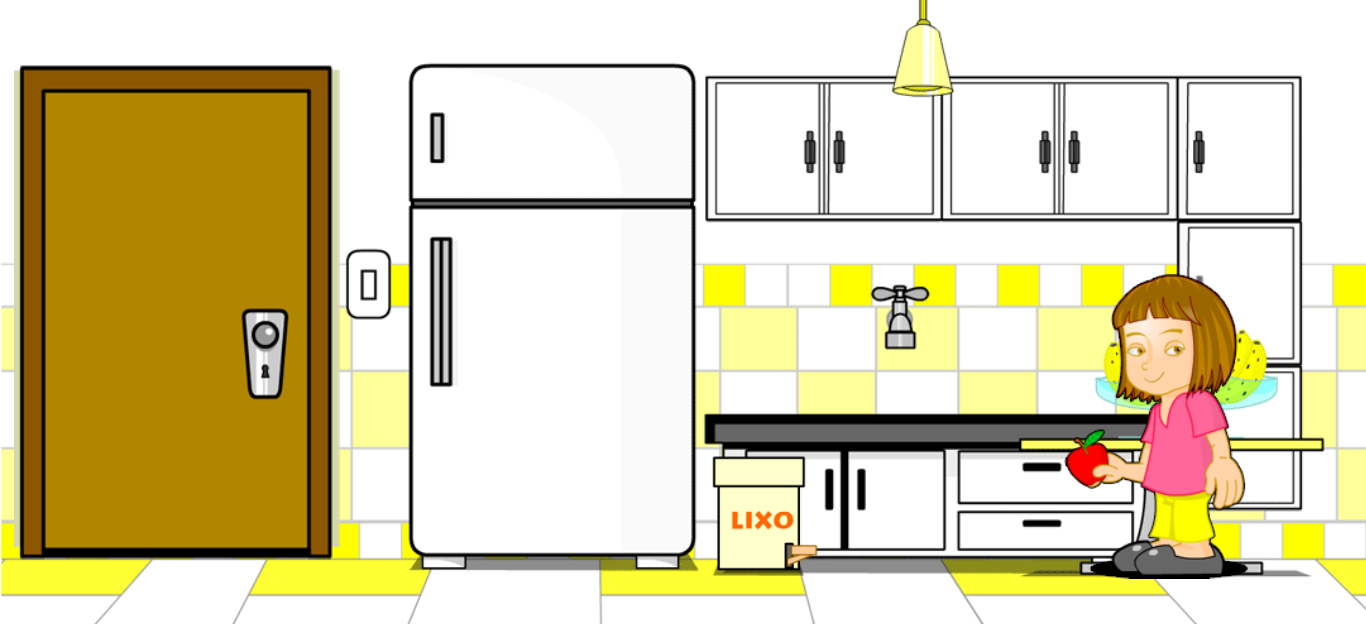


Figure 28: Kitchen screen with a child washing an apple.


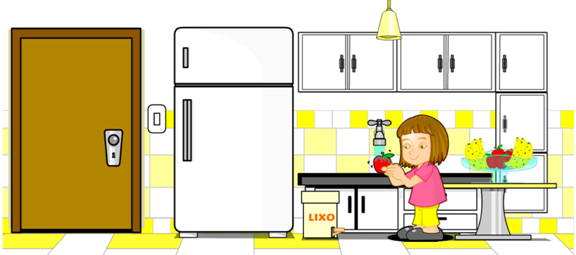


Figure 29: Kitchen screen with a child eating an apple.


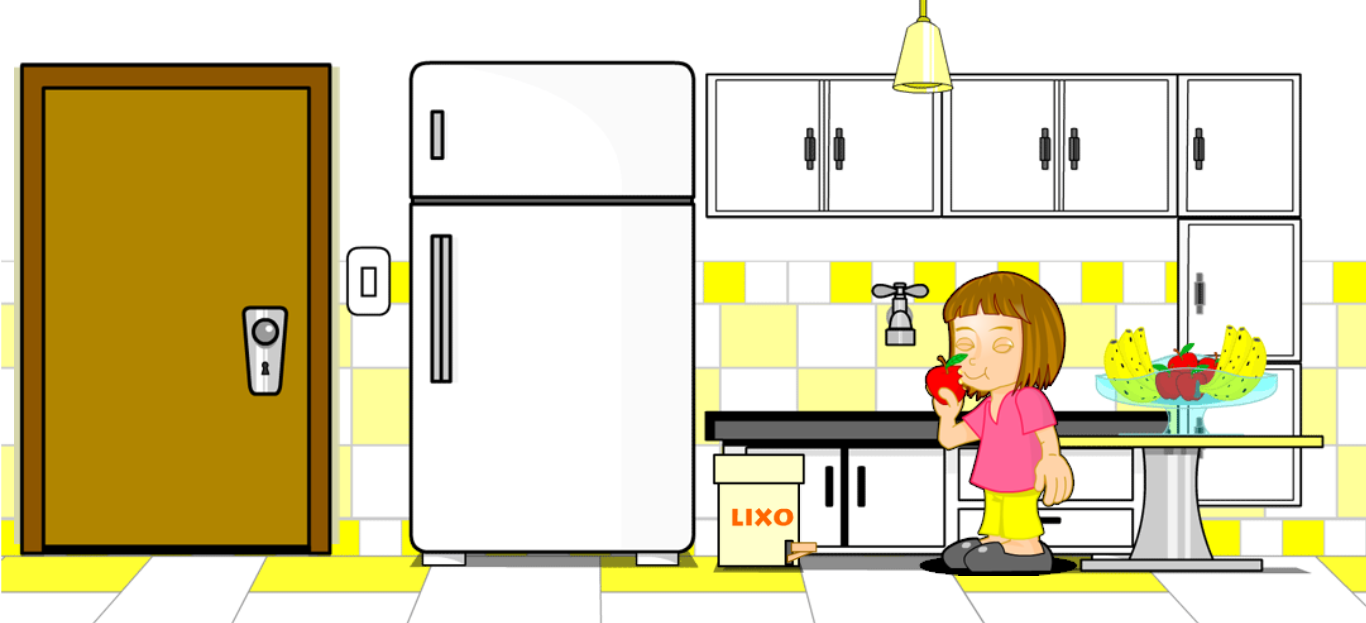


Figure 30: Kitchen screen with a child throwing the rest of the apple in the trash.


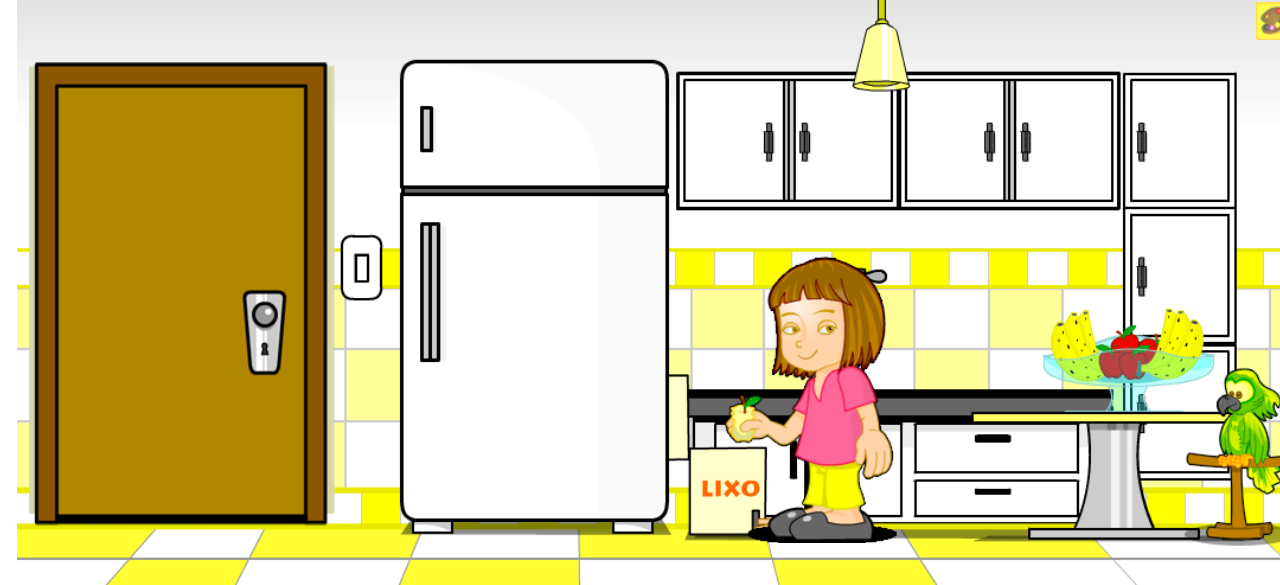


Figure 31: Kitchen screen with child holding the peeled banana.


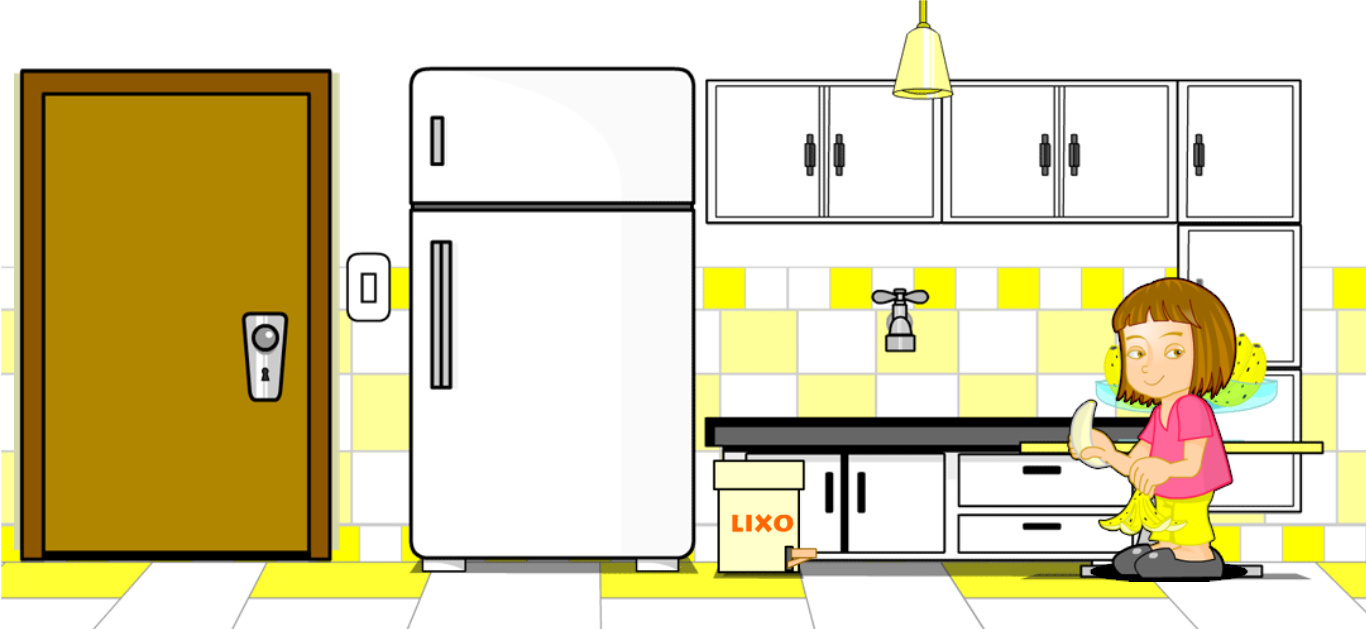


Figure 32: Kitchen screen with child throwing away the peel.


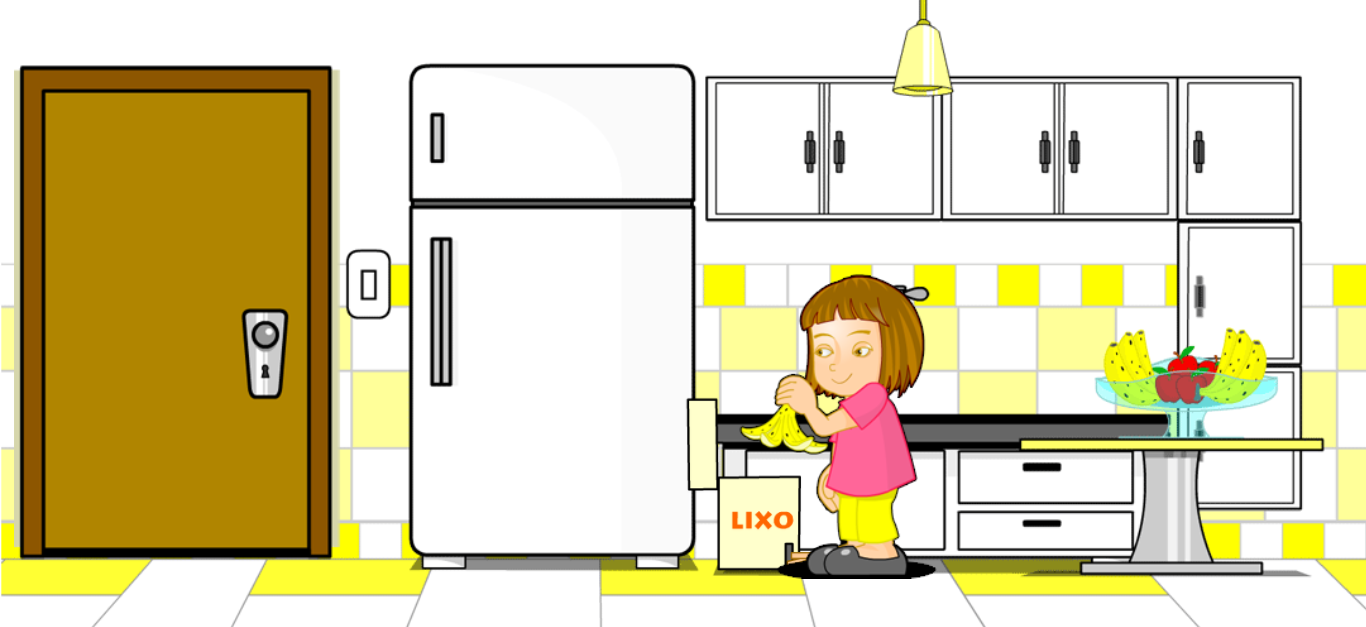


Figure 33: Kitchen screen with child picking the snack in the refrigerator.

**
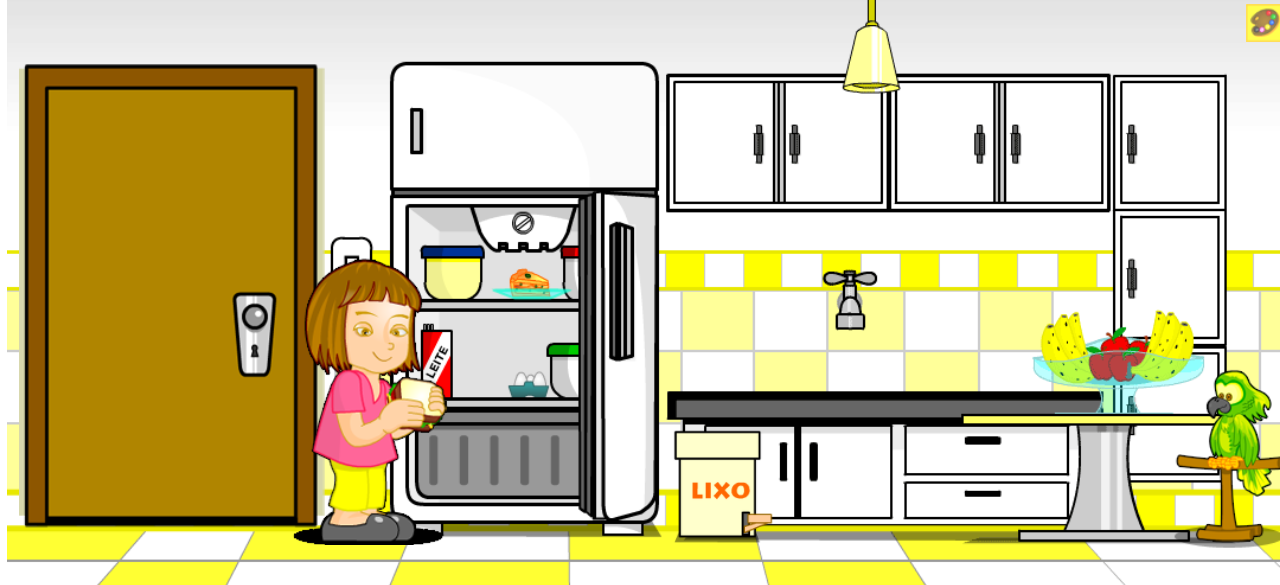
**

Figure 34: Kitchen screen with child eating a snack

**
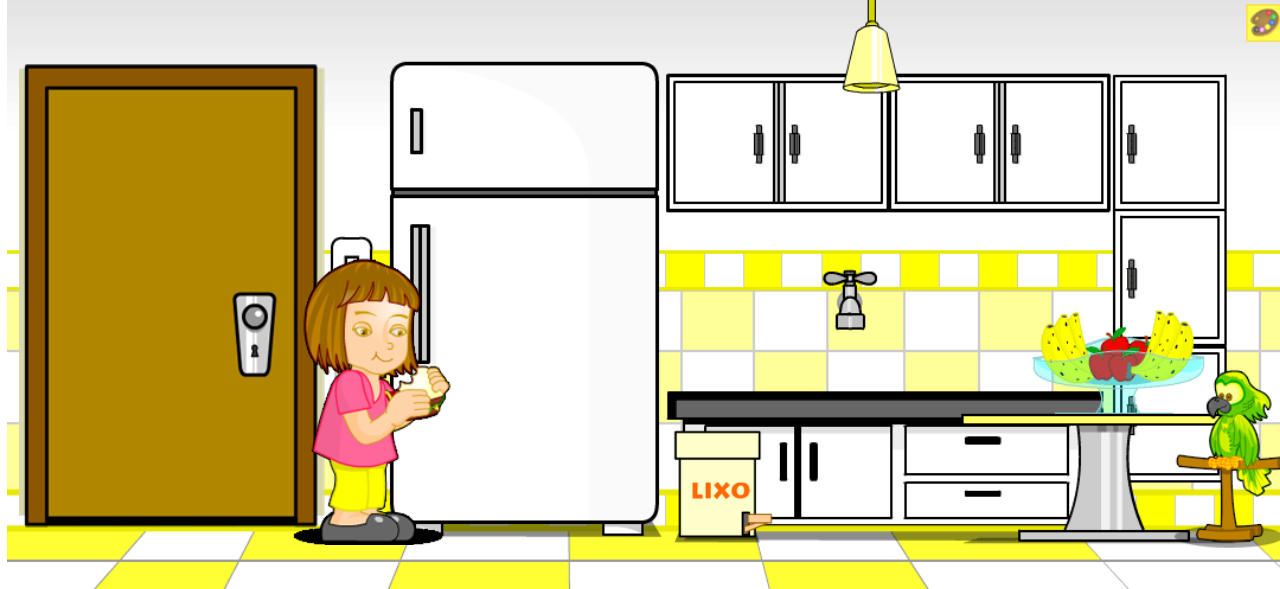
**

- **Bathroom Routine**

In the BATHROOM environment the child must perform the procedure to brush the teeth and in the beginning the mother appears informing what should be done as shown in Figures 35 to 39.

Figure 35: Instruction of what should be done in the bathroom.


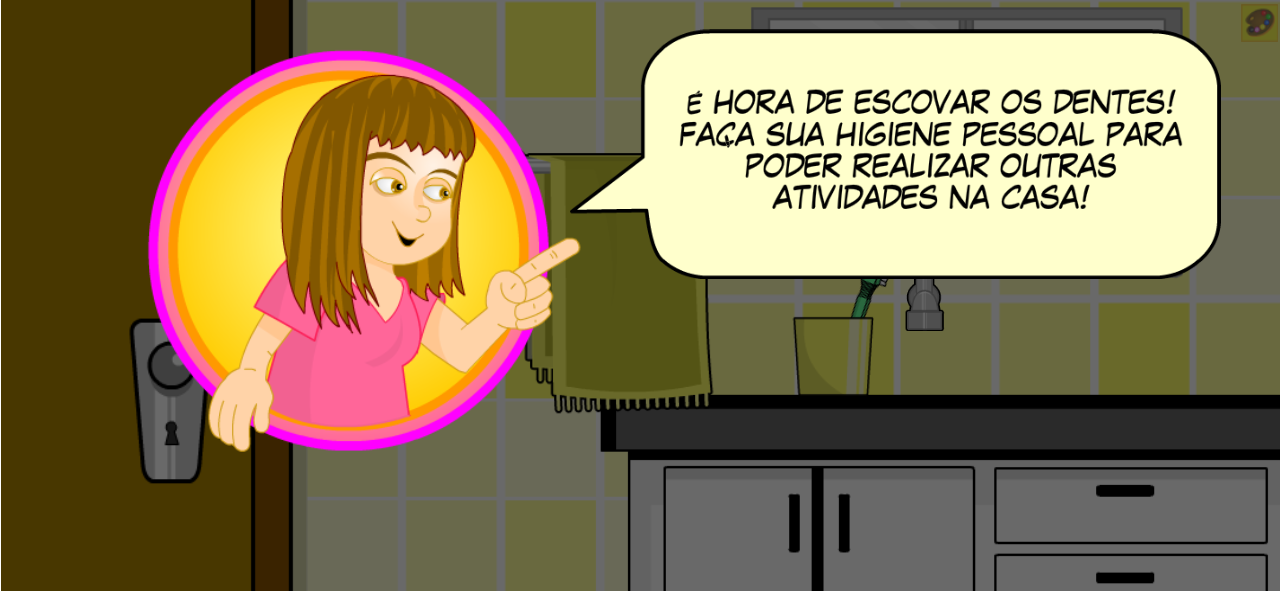


It’s time to brush your teeth! Do your personal hygiene so then you can do the other activities in the house!

**Figure 36:** Bathroom screen with the lights off


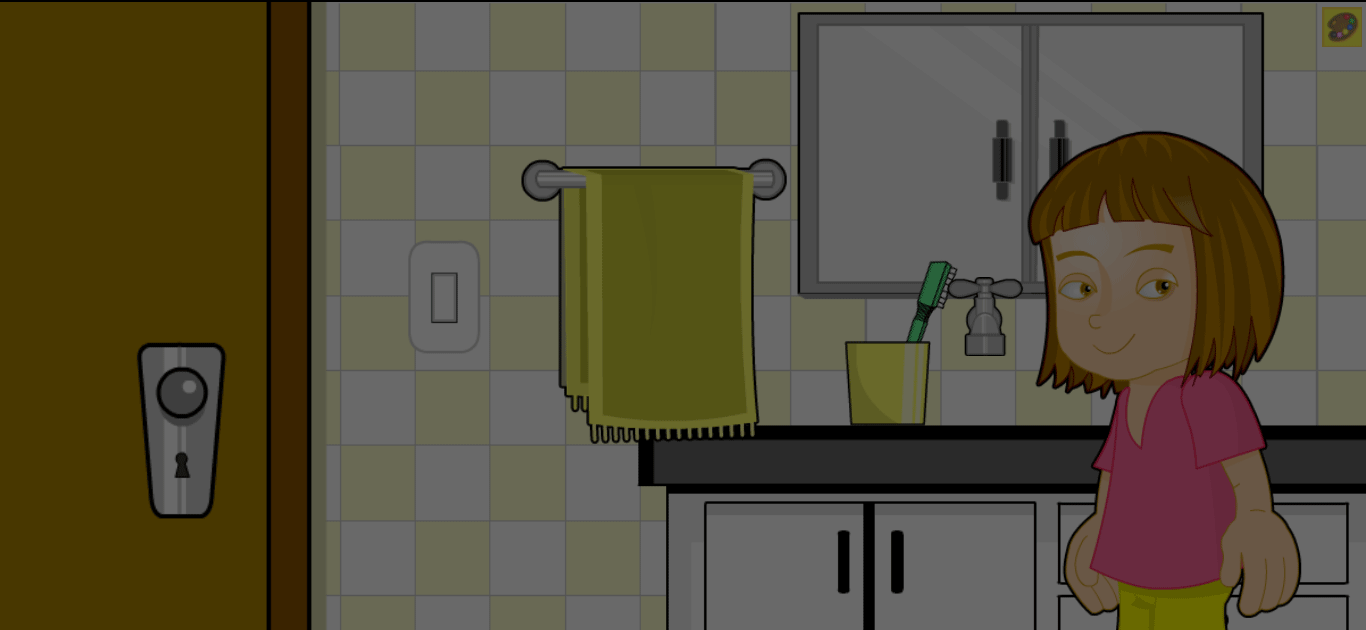


Figure 37: Bathroom screen of the child washing her hand.


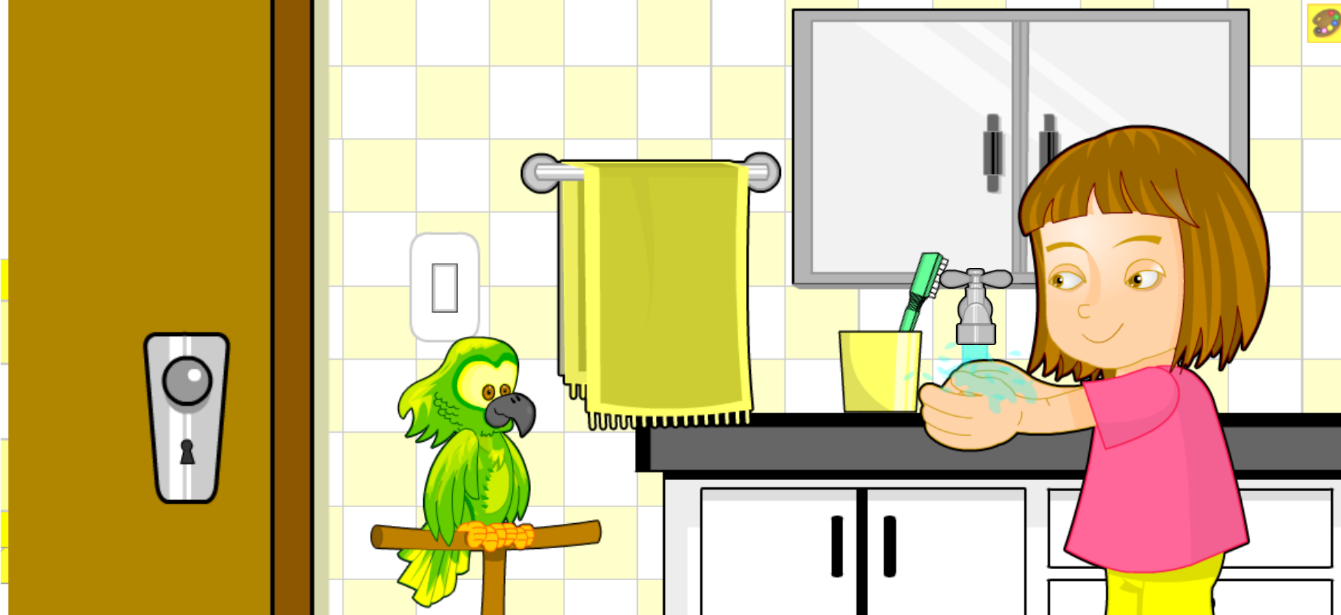


Figure 38: Screen with child brushing her teeth.


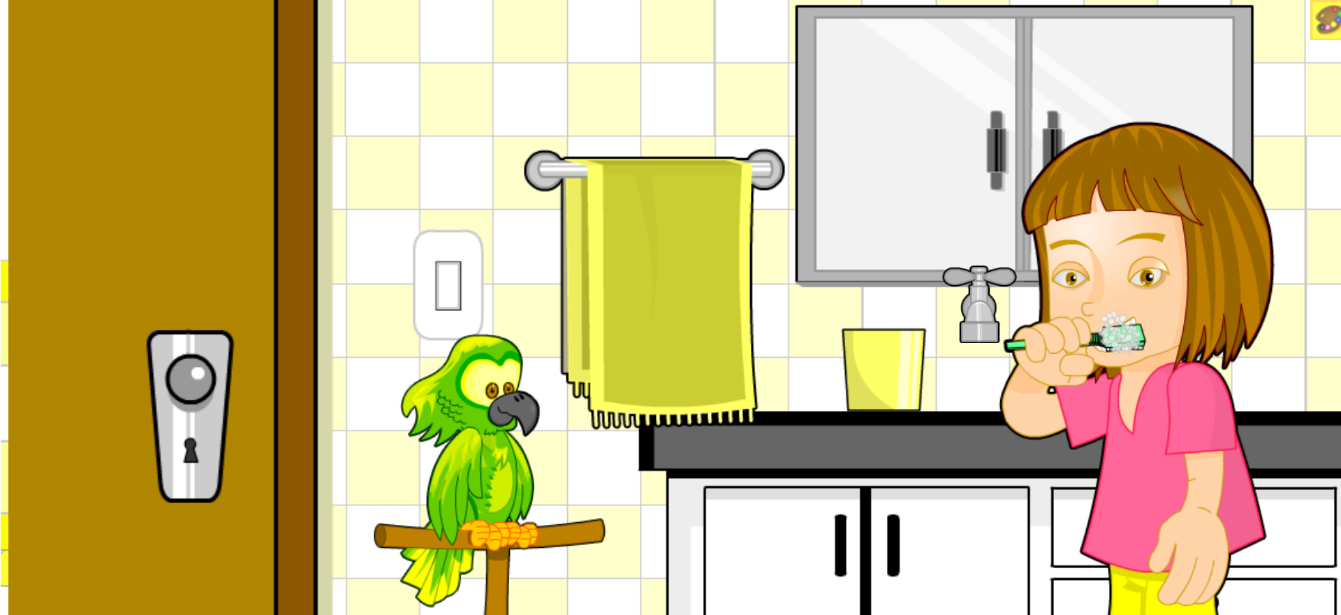


Figure 39: Bathroom screen with child wiping hands on towel.


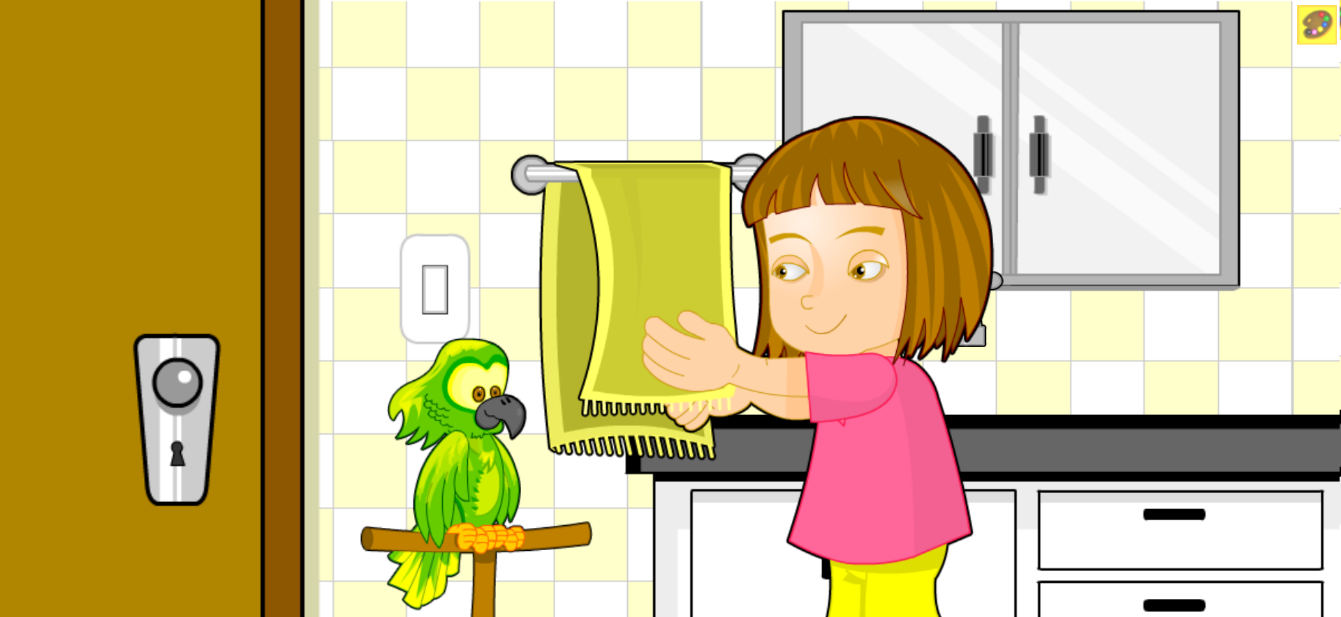


- **Living Room Routine**

In the Living Room environment it will be clear to the child that she cannot feed herself in an environment other than the kitchen and will have fun with nine television channels, being able to interact between the channels and change the volume whenever she wants and before these activities begin the mother will appear saying what should be done, as shown in Figures 40 to 44.

Figure 40: Instructions of what should be done in the living room.


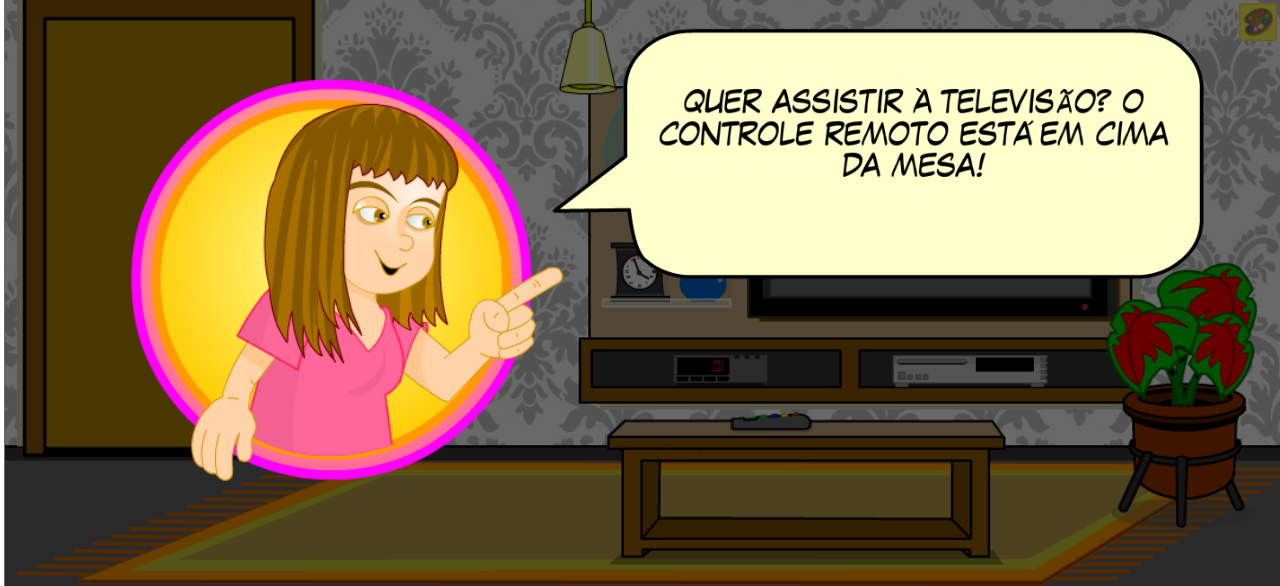


Want to watch TV? The remote control is on the table!

Figure 41: Living room with the lights off


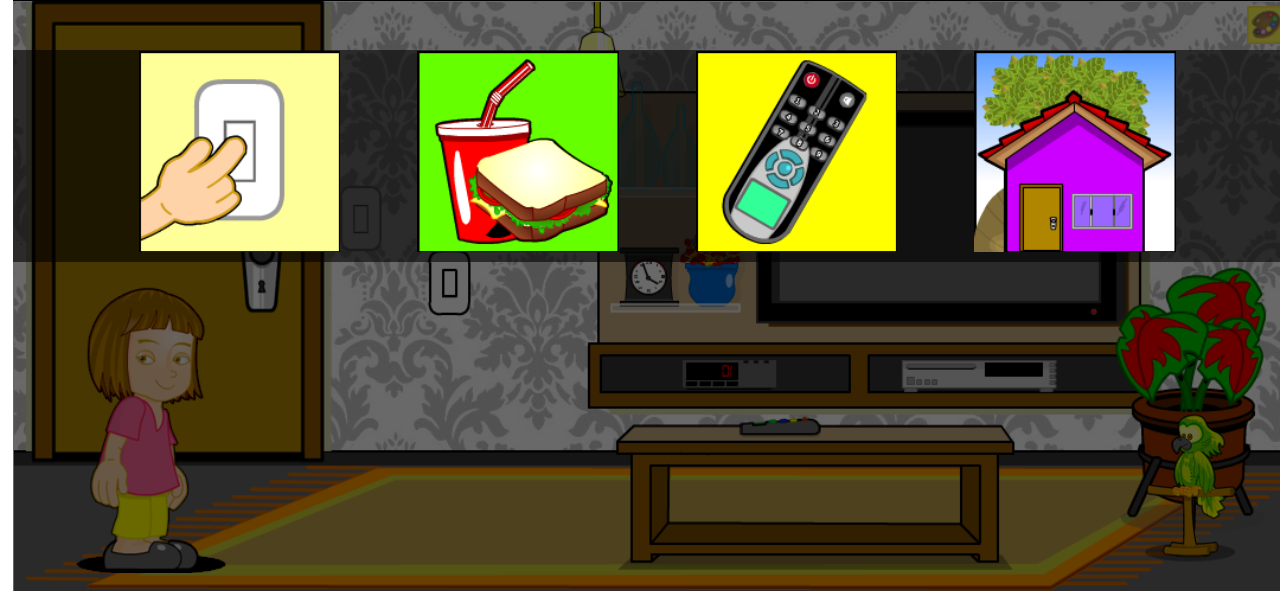


Figure 42: Screen with living room light on


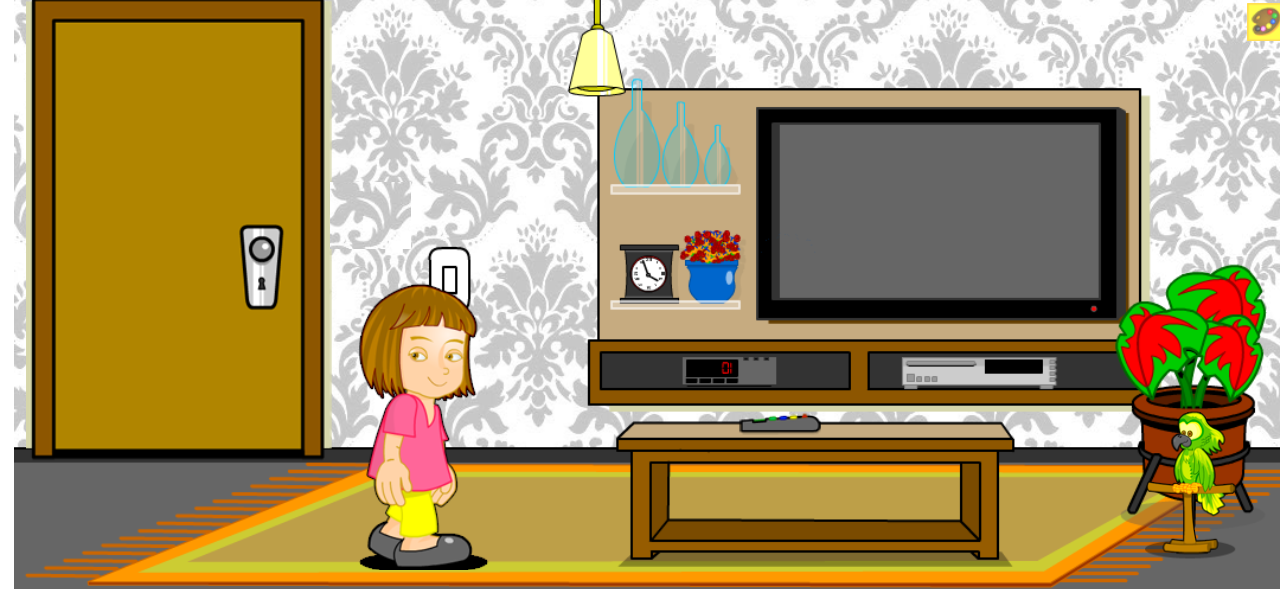


Figure 43: Screen with picture of the TV and remote control where the person can become dependent on TV and watch the channels.


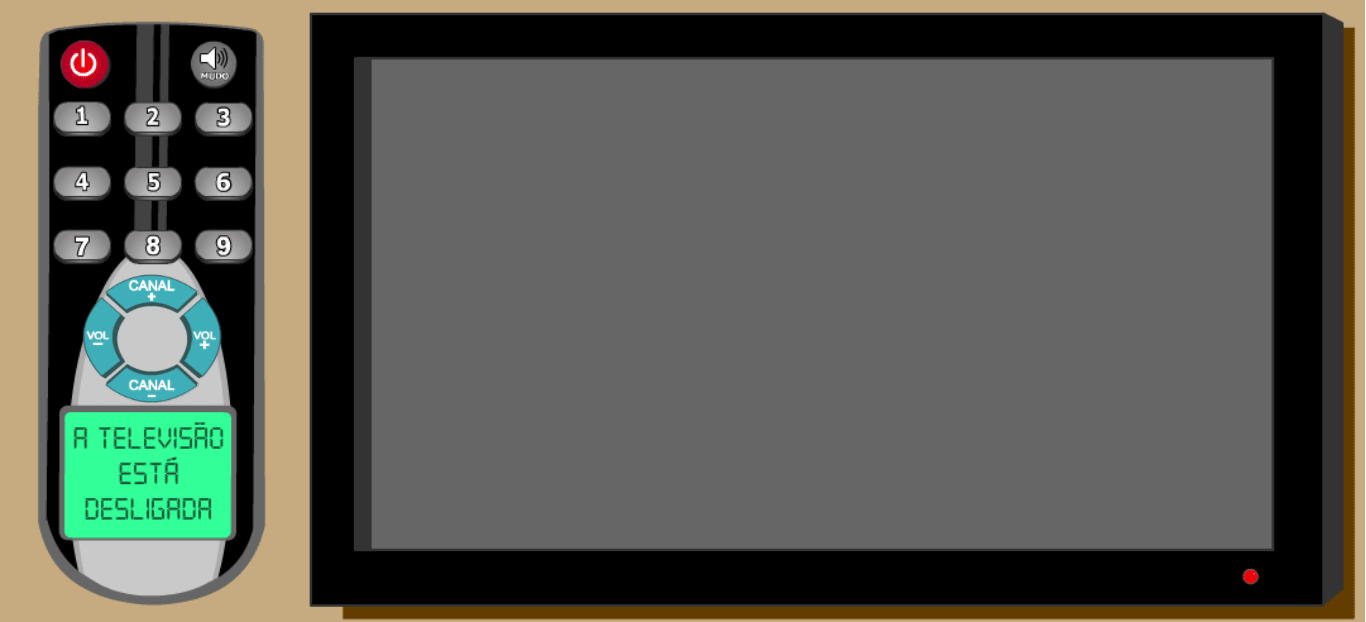


Figure 44: TV screen displaying channel 4 (the game has 9 different channels for the child to watch).


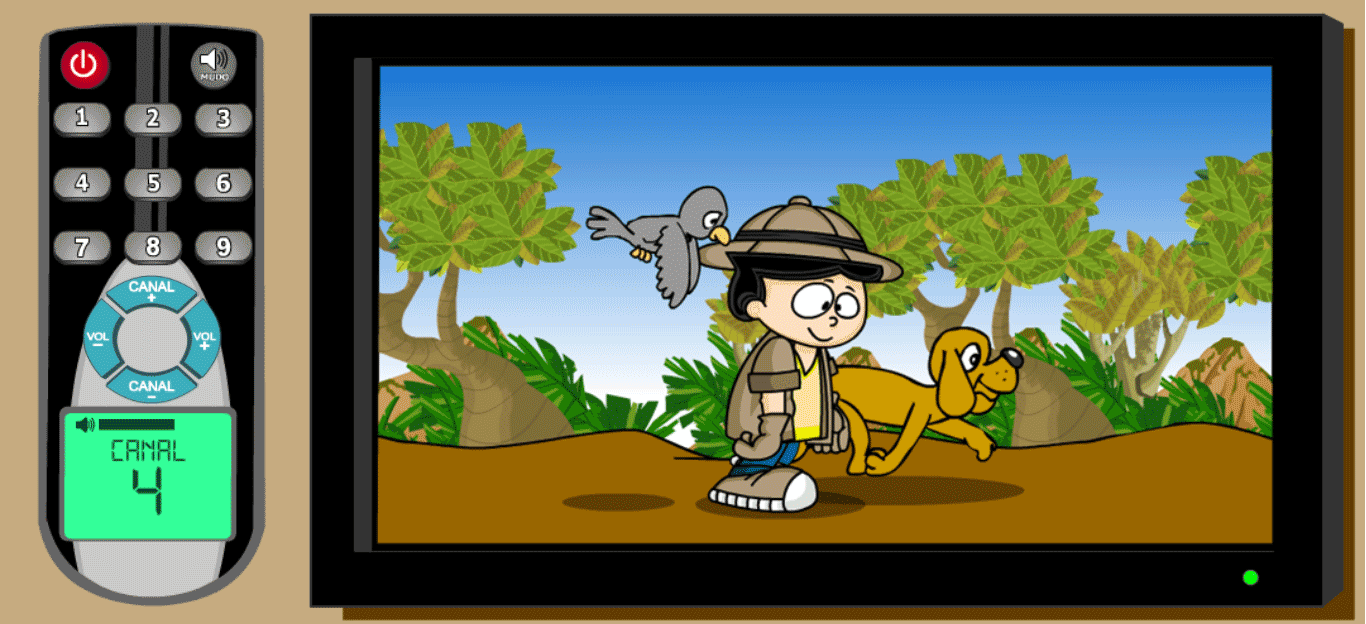


- **Swimming Pool Routine**

In the POOL environment, the child can have fun and show how important the use of the float is. In addition, when entering the environment, the mother will say what should be done, as shown in Figures 45 to 48.

Figure 45: Instruction of what to do in the pool


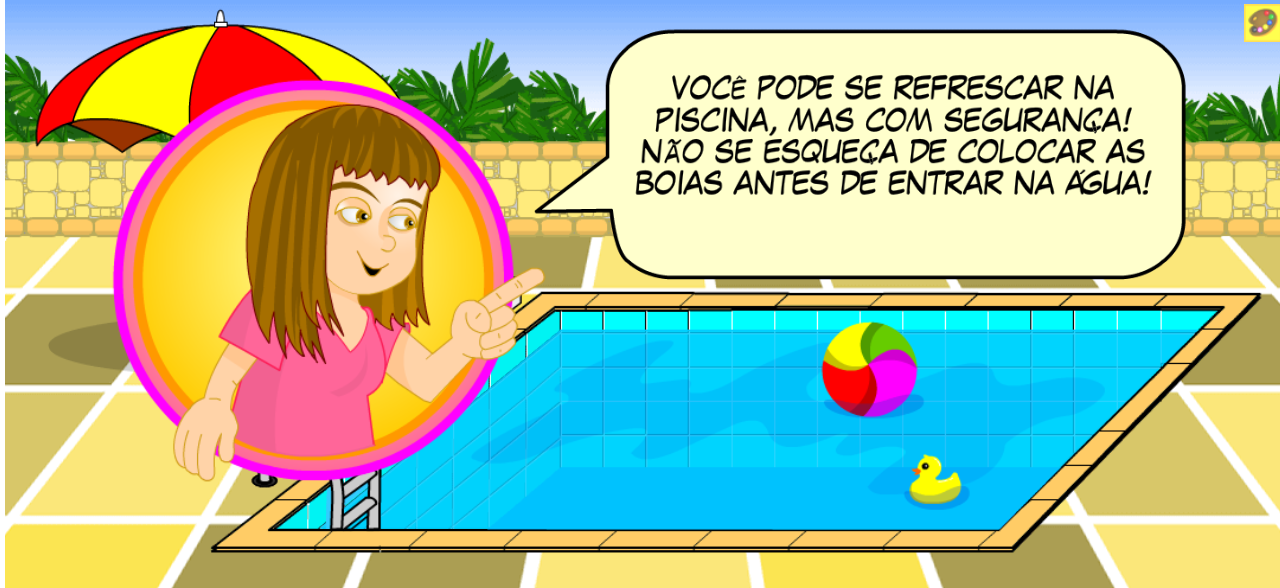


You can enter the swimming pool, but with safety! Don’t forget to put on floats before entering the water!

Figure 46: Pool screen with toys


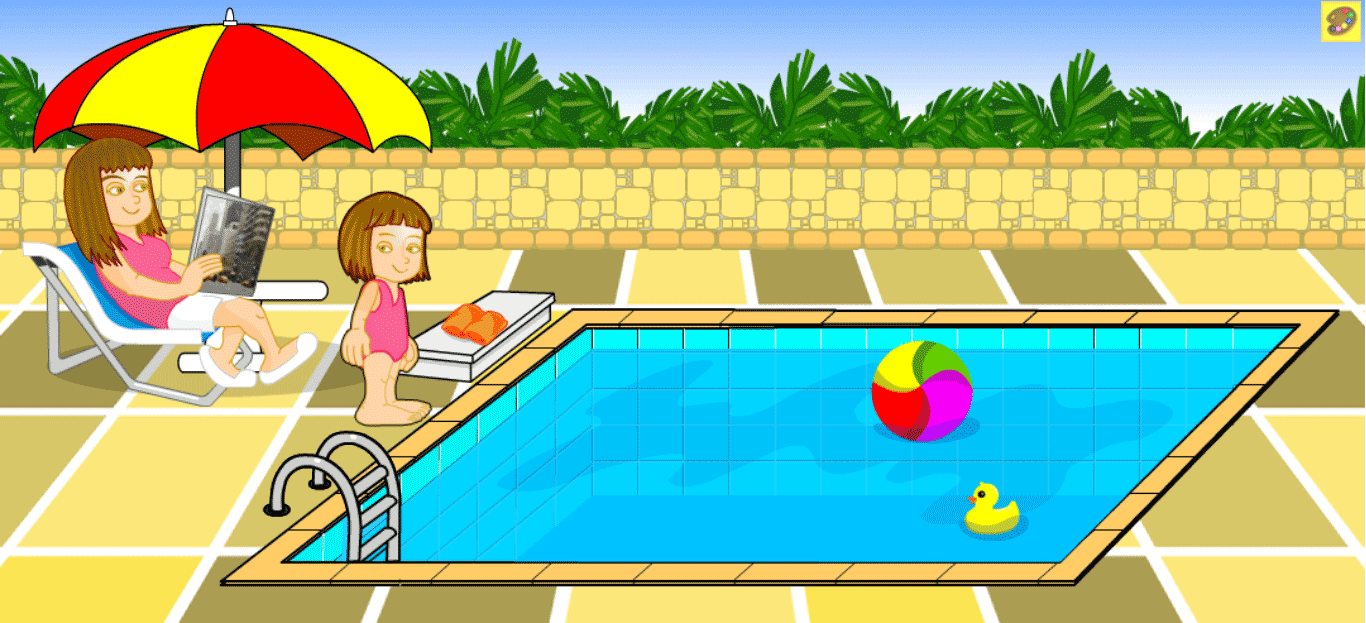


Figure 47: Pool screen with child swimming.


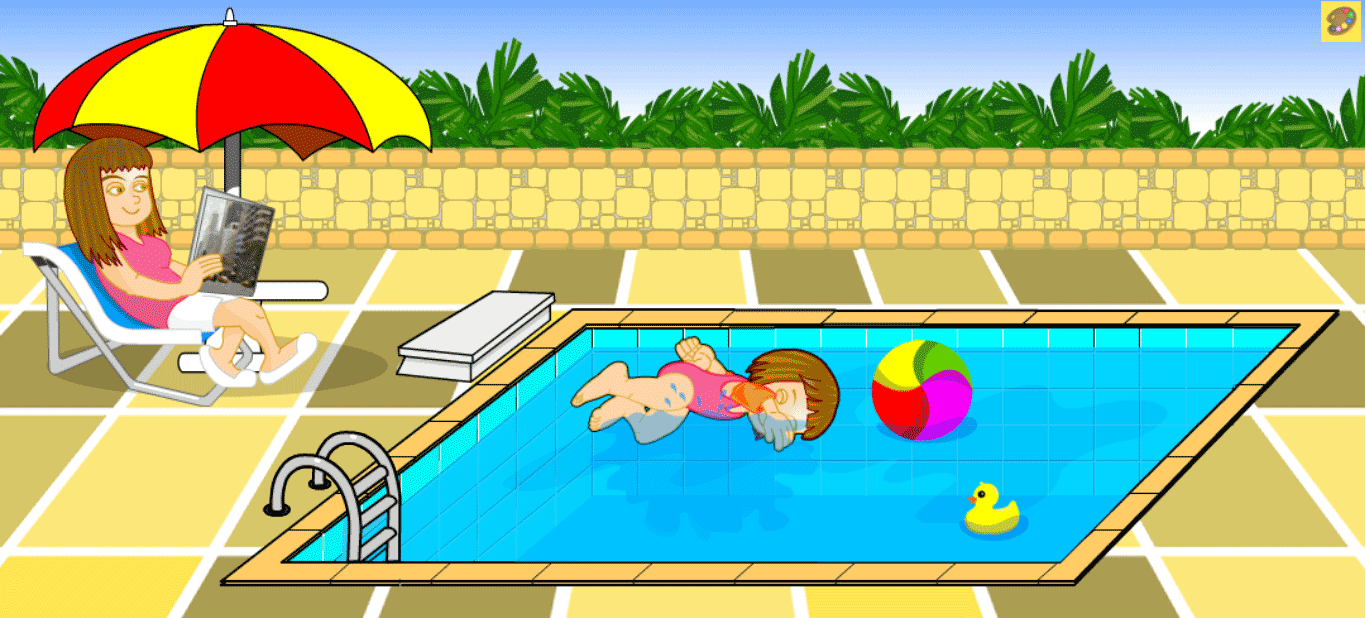


Figure 48: Pool screen with child climbing the stairs


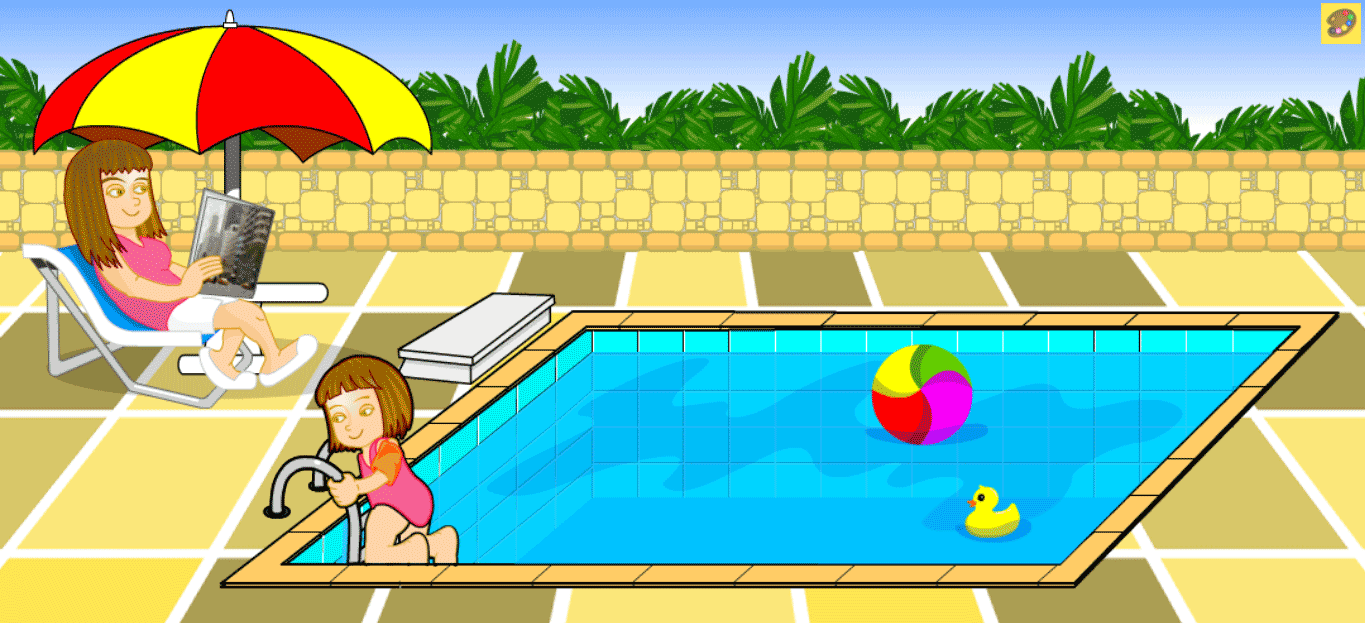

Supplement: Supplementary file 1 — Supplementary material. [file 41598_2020_60014_MOESM1_ESM.docx]
